# A biogeographic framework of octopod species diversification: the role of the Isthmus of Panama

**DOI:** 10.7717/peerj.8691

**Published:** 2020-03-27

**Authors:** Francoise D. Lima, Jan M. Strugnell, Tatiana S. Leite, Sergio M.Q. Lima

**Affiliations:** 1 Department of Botany and Zoology, Universidade Federal do Rio Grande do Norte, Natal, Rio Grande do Norte, Brazil; 2 Centre for Sustainable Tropical Fisheries and Aquaculture, James Cook University, Townsville, Queensland, Australia; 3 Department of Ecology and Zoology, Universidade Federal de Santa Catarina, Florianópolis, Santa Catarina, Brazil

**Keywords:** Phylogeny, Vicariance, Dispersal, Speciation, Fossil calibration, Octopus, Isthmus of Panama, Evolution

## Abstract

The uplift of the Isthmus of Panama (IP) created a land bridge between Central and South America and caused the separation of the Western Atlantic and Eastern Pacific oceans, resulting in profound changes in the environmental and oceanographic conditions. To evaluate how these changes have influenced speciation processes in octopods, fragments of two mitochondrial (Cytochrome oxidase subunit I, COI and 16S rDNA) and two nuclear (Rhodopsin and Elongation Factor-1α, EF-1α) genes were amplified from samples from the Atlantic and Pacific oceans. One biogeographical and four fossil calibration priors were used within a relaxed Bayesian phylogenetic analysis framework to estimate divergence times among cladogenic events. Reconstruction of the ancestral states in phylogenies was used to infer historical biogeography of the lineages and species dispersal routes. The results revealed three well-supported clades of transisthmian octopus sister species pair/complex (TSSP/TSSC) and two additional clades showing a low probability of species diversification, having been influenced by the IP. Divergence times estimated in the present study revealed that octopod TSSP/TSSC from the Atlantic and Pacific diverged between the Middle Miocene and Early Pliocene (mean range = 5–18 Ma). Given that oceanographic changes caused by the uplift of the IP were so strong as to affect the global climate, we suggest that octopod TSSP/TSSC diverged because of these physical and environmental barriers, even before the complete uplift of the IP 3 Ma, proposed by the Late Pliocene model. The results obtained in this phylogenetic reconstruction also indicate that the octopus species pairs in each ocean share a recent common ancestor from the Pacific Ocean.

## Introduction

The formation of the Isthmus of Panama (IP) caused profound changes in environmental and oceanographic conditions (Haug & Tiedemann, 1998; Bartoli et al., 2005; Schneider & Schmittner, 2006), which influenced dispersal and speciation processes in terrestrial and marine biota (Lessios, 1981; Knowlton & Weigt, 1998; Leigh, O’Dea & Vermeij, 2013). The closure of the connection between the Atlantic and Pacific oceans is considered the most important vicariant event of the Cenozoic (O’Dea et al., 2016), providing a remarkable system to study evolutionary processes in a natural environment.

The age of the final closure of the IP, as well as its role in fundamental evolutionary processes is controversial (Stone, 2014), and two hypotheses were proposed to describe this event: the Late Pliocene and the Middle Miocene models. Using geological, fossil and molecular data, several authors have proposed that the final closure of the isthmus was in the Late Pliocene, approximately 2.5–3.5 millions years ago (Ma) (Coates et al., 1992; Bartoli et al., 2005; Schneider & Schmittner, 2006; Coates & Stallard, 2013; O’Dea et al., 2016). However, recent studies based on dispersal waves of terrestrial organisms and geochronological information have suggested that the closure of the this seaway occurred during the Miocene (15 Ma) (Bacon et al., 2015; Hoorn & Flantua, 2015; Montes et al., 2015).

According to the Late Pliocene hypothesis, this long process initiated with a collision of Central and South America about 15–24 Ma ago and the formation of a volcanic arc, around the Early Miocene (Coates & Stallard, 2013). During the Middle Miocene (around 10 Ma), successive collisions caused widespread shallowing of the oceans and major changes of oceanic conditions, and deep and intermediate water exchanges between the Atlantic and Pacific were closed (Coates et al., 2004; Keigwin, 1982). Around 6–4 Ma oceanic conditions, including temperature, salinity, sedimentary carbon content and habitat availability on each side of the isthmus changed substantially (Haug & Tiedemann, 1998; Leigh, O’Dea & Vermeij, 2013). By 3 Mya, the uplift of the IP completely separated the waters of the Tropical Western Atlantic and the Tropical Eastern Pacific (Coates & Stallard, 2013; O’Dea et al., 2016).

Studies based on Uranium-lead geochronology in detrital zircons in the Andes, Panamanian fluvial deposits and inferences on terrestrial and aquatic dispersal provide a different insight for the early closure of the IP; the Middle Miocene hypothesis (Montes et al., 2012; Bacon et al., 2013; Bacon et al., 2015; Montes et al., 2015). In this approach, the isthmus formation started around 38–28 Ma, and the collision between the southern tip of Central America and South America occurred during the Late Oligocene (28.1–23.0 Ma). Montes et al. (2015) suggested that the complete closure interrupting the water connection between the Eastern Pacific and Western Atlantic occurred around 14–15 Ma.

The timing of the uplift of the IP, based mainly on the Late Pliocene hypothesis, is widely used as a biogeographical calibration point and is considered one of the most important geological events for calibrating molecular clocks (Lessios, 2008; Parham et al., 2012; Gleadall, 2013). However, to evaluate the influence of the final seaway closure on divergence and distribution of Atlantic and Pacific sister species, it is important to use a calibration independent of the isthmus formation to avoid circular reasoning in the divergence time interpretation (Marko, 2002; O’Dea et al., 2016). Therefore, molecular phylogenies containing sister species from each side of the IP have been calibrated using the fossils record and/or the molecular evolutionary rate of a particular gene (e.g., Bermingham & Lessios, 1993; Knowlton et al., 1993; Collins, 1996; Bartoli et al., 2005; Bacon et al., 2013; Galván-Quesada et al., 2016).

The emergence of geographical barriers, such as the formation of the Isthmus of Panama, may reduce or interrupt gene flow between populations. The vicariant populations can evolve different genotypic characteristics over time, although they can retain similar morphological and behavioural characteristics (Palumbi, 1994; Knowlton, 2000; Cowman & Bellwood, 2013). The species that diverged as a consequence of the uplift of the Isthmus of Panama are closely related morphologically and genetically and are called transisthmian sister species pairs/complex (TSSP/TSSC) (Lessios, 2008; Marek, 2015; Marko, 2002). According to the transisthmian sister species complex concept, several species on one side of the Isthmus can represent the putative sister group of the species on the other side (Marek, 2015).

Transisthmian clades including isopods, echinoids, crustaceans, fishes and molluscs have already been identified on each side of the Isthmus of Panama based on molecular and fossil calibrated phylogenies (Graham, 1971; Lessios & Weinberg, 1994; Lessios, 2008; Marko, 2002; O’Dea et al., 2016). Nesis (2003) stated that there are no cephalopod species occurring on both sides of the IP, although nine pairs of similar species are known in the Eastern Pacific and Western Atlantic: two pairs of shallow-water squids (Family: Loliginidae) and seven pairs of benthic octopuses (Family: Octopodidae). According to Voight (1988), the high degree of similarity among pairs of pygmy, ocellated, common and striped octopod species, distributed along either side of Central America, is evidence that each pair shares a common ancestor, which is a more parsimonious explanation than convergent evolution.

The Superfamily Octopodoidea includes the families Octopodidae, Bathypolypodidae, Enteroctopodidae and Megaledonidae, which encompass a high diversity of benthic octopus (Boyle & Rodhouse, 2005; Norman, Hochberg & Finn, 2014; Sanchez et al., 2018). Some species have a planktonic phase before settling on the substrate and are characterised by high fecundity (20,000–500,000 eggs) and small eggs (20–50 mm). Other species have low fecundity (50–800 eggs), large eggs (6–18 mm) and hatchlings that settle directly on the seabed (Mangold, 1987; Iglesias et al., 2007). The oceanographic characteristics such as temperature, ocean currents and productivity are important determinants of trait evolution of larvae and their dispersal ability (Robertson & Collin, 2015). For this reason, the contrasting life history traits among octopod species make them an interesting model to study the processes of speciation and adaptive divergence after environmental changes caused by a vicariant event.

The consequences of forming the Isthmus of Panama were dramatic to marine biodiversity, ocean circulation and global climate. Thus, this study aims to understand how the uplift of the IP and the environmental changes caused by this vicariant event influenced speciation and dispersal processes of octopod species in the Atlantic and Pacific oceans. Based on that, Bayesian time-calibrated (biogeographical and fossil) phylogenetic analysis was performed to identify putative transisthmian sister species pairs/complexes and verify whether the divergence time among the octopod lineages coincides with the Pliocene or Miocene hypotheses.

## Material & Methods

### Data collection

**Figure 1:**
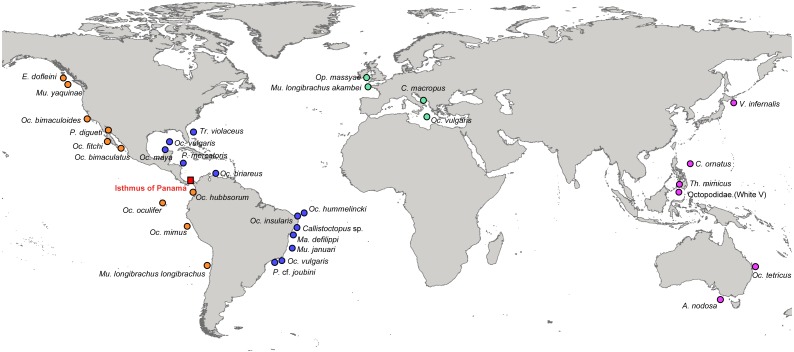
Localities of the specimens used in this study to estimate phylogenetic relationships and divergence times among octopods species. Orange circles represent species from the Eastern Pacific (EP), blue circles from the Western Atlantic (WA), green circles from the Eastern Atlantic (EA), and pink circles are species from the Western Pacific (WP). The Isthmus of Panama is indicated with a red square.

Tissue samples of octopod species were collected by snorkelling and SCUBA diving in Brazil (North-eastern coast and four oceanic islands) (permit SISBIO 10706-5 and 30484-1), from fish markets and landings in Mexico (Isla Mujeres, Sisal and port of Progreso) (Fig. 1) (Table S1). Muscle tissue samples were taken from octopod arms preserved in 95% ethanol and stored at −20°C.

Initial analyses were performed using sequences generated in the present study, and additional sequences were obtained from GenBank in order to evaluate the closest phylogenetic relationships among putative octopods sister species occurring in the Atlantic and Pacific. A total of 135 sequences from 30 cephalopods species (70 from this study and 65 obtained from GenBank) were chosen to estimate divergence times and infer phylogenetic relationships. The sequences of genes fragments generated in this study are accessible from GenBank under accession numbers (COI: MN933632 –MN933651; 16S: MN508063 –MN508082; rhodopsin: MN946381 –MN946396; EF-1α: MN946371, MN946372, MN946373, MN946374, MN946375, MN946376, MN946377, MN946378, MN946379, MN946380) (Table S1).

### Calibration priors

Four fossils and also biogeographical information (Table 1) were used in order to calibrate the species tree. Calibration priors used include:

**Table 1:** Details of fossil and biogeographical information used to calibrate the phylogeny. The prior probability distributions and posterior probability densities after the Monte Carlo Markov Chain (MCMC) run are showed.

**Calibration node**	**Distribution**	**Type**	**Prior (Ma)**			**MCMC results (Ma)**	
			**Mean**	**Offset**	**95% CI**	**Mean**	**95% HPD**
*M. longibrachus akambei*×*M. longibrachus longibrachus*	Normal	Biogeographical	0.025	–	0.015, 0.035	0.032	0.024, 0.04
*Argonauta nodosa*×*Tremoctopus violaceus*	Exponential	Fossil	35	29	30, 62	46	29, 68
Cirrata × Incirrata	Exponential	Fossil	25	90	95, 187	101	90, 121
Vampyromorpha × Octopoda	Exponential	Fossil	24	162	162, 250	170	162, 187

**Notes.**

Ma millions of years ago CI confidence interval HPD Highest Posterior Density

1—The biogeographic prior was the separation of deep sea *Muusoctopus longibrachus* subspecies (*Muusoctopus longibrachus longibrachus* Ibáñez, Sepúlveda & Chong, 2006 and *Muusoctopus longibrachus akambei* Gleadall et al., 2010) as a result of events related to the Last Glacial Maximum (LGM) proposed by Gleadall (2013). A normal prior was set on the *M. longibrachus* node with a mean of 24 ± 5 kya, yielding a range between 5% and 95% quartiles of 15–33 kya (Gleadall, 2013).

2—Divergence between *Argonauta* and *Tremoctopus*. An exponential prior was used based on the earliest record of the Argonautidae, the fossil *Obinautilus pulcher* from the Oligocene (29 Ma) (Kobayashi, 1954). The upper bound of 64 Ma was placed at the last occurrence of ammonites as a prior 95% confidence interval (CI) of the distribution of *Argonauta*. In the Cretaceous, ammonite shells were abundant but were not recorded in the Tertiary. According to Strugnell et al. (2008), although ammonites and argonauts have similar shells, there are no *Argonauta* fossil records as old as ammonite records. Therefore, it is conservative to set this upper bound on the prior 95% CI of the distribution of argonauts at the last occurrence of ammonites, 36 million years prior to their appearance in the fossil record.

3—Split between the suborders Cirrata (*Opisthoteuthis massyae*) and Incirrata based on the fossils of *Keuppia levante*, *K. hyperbolaris* and *Styletoctopus annae* from the Upper Cenomanian, between the Toarcian (180 Ma) and the early Turonian (95 Ma). These fossils are regarded as the earliest representatives of the Incirrata (Fuchs, Bracchi & Weis, 2009). This is represented by an exponential prior on the MRCA of these groups, with the minimum age of 95 Ma and 180 Ma as an upper bound on the prior 95% confidence interval.

4—Separation of Vampyromorpha (*Vampyroteuthis infernalis*) and Octopoda. An exponential prior with lower bound of 162 Ma was chosen based on the fossil *Vampyronassa rhodanica* from the Lower Callovian of the Jurassic (Fisher & Riou, 2002). The upper bound of 250 Ma was based on studies of Strugnell et al. (2006) and Kröger, Vinther & Fuchs (2011), who affirm that Vampyropods diverged by or before the Permian.

### Phylogenetic analysis

Genomic DNA was extracted using the GF-1 Nucleic Acid Extraction kit (Vivantis, Malaysia) according to the manufacturer’s instructions. Fragments of two mitochondrial (Cytochrome oxidase subunit I, COI and 16S rDNA) and two nuclear (Rhodopsin and Elongation Factor-1α, EF-1α) genes were amplified in this study. Cytochrome oxidase I gene amplicons were obtained using universal primers LCO1490 and HCO2198 (Folmer et al., 1994), and partial sequences of 16S rDNA were amplified with the primers 16SarL and 16SbrH (Palumbi, 1996). The forward and reverse primers for amplification of Rhodopsin (RhFwd1 5′ GATCGTTACAATGTCATCGGTAGACC 3′, RhRev4 5′ GAGAAAGAATGCGAAGATGCTA 3′) and EF-1α (EFFwd1 5′ TCTGGTTGGCATGGTGATAACATG 3′, EFRev3 5′ ATTGTCATTAACCACCCTGGAC 3′) were designed from octopus sequences available on GenBank using the software Geneious 9.0.2 (Kearse et al., 2012).

The PCR amplification reactions of all sequences were conducted in a final volume of 25 µL containing 1 µL forward primer, 1 µL reverse primer (10 mM), 12.5 µL Taq DNA Polymerase Master Mix (Ampliqon A/S) or MyTaq RedMix (Bioline), 8.5 µL H_2_O and 2 µL DNA (20–40 ng/µl). PCR cycle parameters used to amplify COI and 16S genes were 3 min at 95 °C for denaturation, followed by 35 cycles of 1 min at 94 °C, 1 min at 45 °C for annealing, 1.5 min at 72 °C for extension and a final extension step of 4 min at 72 °C. The parameters used to amplify Rhodopsin, detailed in Allcock, Strugnell & Johnson (2008), were the same used to amplify EF-1α. The PCR products were purified and sequenced by Macrogen Inc, Seoul, Korea.

Electropherograms were edited with Geneious 9.0.2 (Kearse et al., 2012), and sequences were aligned by ClustalW using Mega 6 (Tamura et al., 2013). Unalignable loop regions of 16S rDNA and gaps of EF1-alpha were removed before analysis using Gblocks software (Castresana, 2000). The substitution model for each gene was chosen on the basis of the hierarchical and Akaike information criterion tests using the software jModeltest (Posada, 2008). The substitution models most suitable for each gene according to both tests were GTR+G (COI), GTR+G+I (16S), and HKY85+G (rhodopsin and EF1-alpha).

Bayesian phylogenetic inference on the subset of sequences was carried out in BEAST 1.8.4 (Drummond et al., 2012). A total of 33 specimens (COI—33, 16S rDNA—32, rhodopsin—27, EF1-alpha—8) were included in subsequent analyses as separate partitions with unlinked substitution models and linked clock and tree models. An uncorrelated lognormal relaxed clock model incorporating Yule speciation-process prior on branching rates was used. Monte Carlo Markov Chain (MCMC) runs were performed for 3 × 10^8^ generations, sampling one tree each 3 × 10^4^ runs. The convergence of MCMC runs, effective sample size and the correct ‘burn-in’ for the analysis were assessed using Tracer v1.6 (Rambaut et al., 2014). A consensus tree accessing the *posteriori* probability values of each clade was generated using TreeAnnotator 1.8.3 (Drummond et al., 2012) and displayed using FigTree 1.4.3.

### Biogeographical analysis

The distribution ranges of the species were divided into four areas: Western Atlantic (WA), Eastern Atlantic (EA), Western Pacific (WP) and Eastern Pacific (EP) (Fig. 1).

Reconstruct Ancestral State in Phylogenies (RASP) software package (Yu et al., 2015) was used to infer historical biogeography of the lineages, based on present-day distributions of cephalopods species (Norman, Hochberg & Finn, 2014). We used the Bayesian Binary Method (BBM) to estimate the probabilities of ancestral ranges by calculating the average probability of presence (1) and absence (0) over all sampled generations of the ancestral species in each area (Yu et al., 2015). Events of vicariance, dispersal and extinction, as well as the route of dispersal on the nodes, were also calculated by BBM model in RASP. The analysis was carried out with the Maximum Clade Credibility Tree (MCCT) from BEAST phylogenetic reconstruction, setting four heated MCMC chains, which run simultaneously for 5 × 10^6^ generations, sampled every 1,000 generations.

The Time-Event Curve (TEC) was obtained by re-calculating the time of each node using the time of the root. The events on the node were treated using a modified Gaussian distribution (Yu et al., 2015). Thus, events of extinction, dispersal and vicariance are assigned to a time frame. This analysis was also carried out using RASP.

All the clades considered as transisthmian must contain species from each side of the IP and must have diverged after a vicariance event indicated by the RASP analysis.

## Results

A total of 1,992 bp of the combined dataset (COI—609, 16S—396, rhodopsin—546, EF—441) were used to infer phylogenetic relationships and divergence times among 30 cephalopod species.

### Phylogenetic analysis

**Figure 2:**
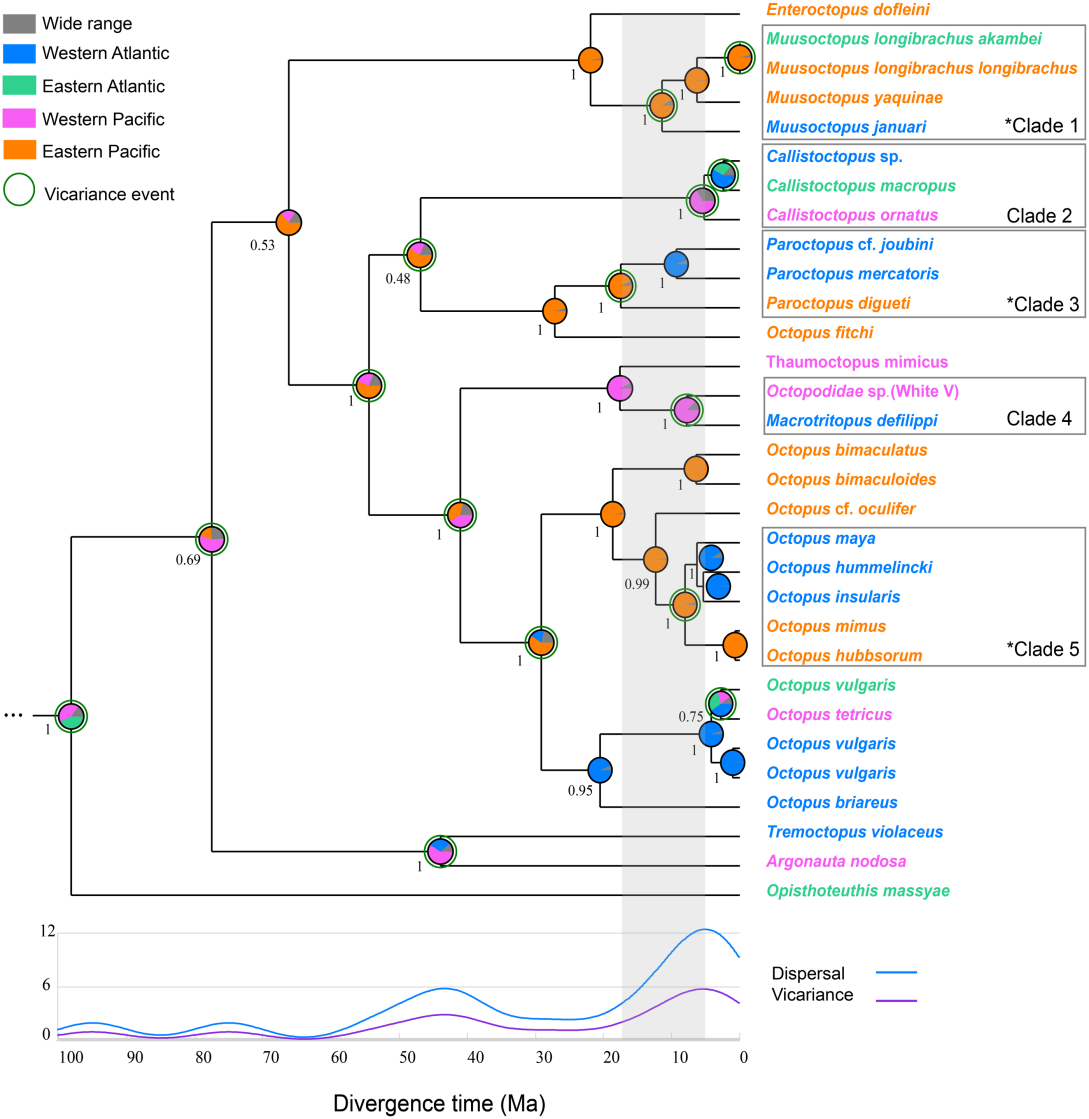
Integrated Bayesian phylogenetic tree and reconstruction of ancestral state. The Bayesian posterior probabilities of the clades are shown below the nodes. Pie charts show the posterior probabilities of ancestral areas on different nodes. The five clades discussed in this study are highlighted in the phylogeny. The transisthmian sister species pair/complex are indicated by an asterisk in the clades 1, 3 and 5. Gray area represents the interval of divergence time found in octopod species (5.22–17.24). The circles surrounding some pie charts on the nodes indicate vicariance events. The graph shows events of dispersal and vicariance (axis y) assigned to a time frame as a result of a modified Gaussian distribution. *Vampyroteuthis infernalis* is not shown here due to its long branch length (see Fig. S1).

The Bayesian phylogenetic tree, built using a relaxed phylogenetic approach, reveals three well-supported clades of transisthmian octopus species pair/complex (clades 1, 3, 5) that underwent vicariance processes as indicated by the RASP analysis (Fig. 2). Two other clades were also recovered, in which the divergence processes among the species showed a low probability of having been influenced by the IP (clades 2 and 4) (Fig. 2). The divergence time estimation indicated that the mean age of separation among TSSP/TSSC varied from 5.22 to 17.24 Ma (Table 2).

Clade 1 is composed by the deep sea *Muusoctopus* species (Family: Enteroctopodidae) and is characterised by two vicariant events. The first occurred 11.39 million years ago (5.08, 20.77 95% HPD) at the divergence of *M. januari* (WA) and a clade containing *M. yaquinae* (EP) and *M. longibrachus* subspecies (Posterior probability [PP] = 1). The second vicariance event corresponds to the divergence of *M. longibrachus longibrachus* and *M. longibrachus akambei*.

Clade 2 comprises the nocturnal species *Callistoctopus ornatus* from WP and *Callistoctopus* sp. and *C. macropus* from WA and EA, respectively (PP = 1). The divergence between those two lineages from Atlantic and Pacific was estimated to occur 5.22 Ma (2.21, 10.96 95% HPD).

**Table 2:** Divergence time estimates for each clade containing transisthmian sister species pair/complex. The events of dispersal and vicariance are shown for each clade. The possible routes of dispersal for the most recent common ancestor in each clade are also shown with their respective probabilities.

**Clade**	**Divergence time (Ma)**		**Vicariance event**	**Dispersal event (N)**	**BBM Route**	**BBM Probability**
	**Mean**	**95% HPD**				
1	11.39	5.08, 20.77	1	2	EP->WA	0.8801
2	5.22	2.21, 10.96	1	2	WP->WA	0.3678
3	17.24	8.99, 30.31	1	2	EP->WA	0.8745
4	7.83	2.82, 15.88	1	2	WP->WA	0.8771
5	8.03	4.27, 13.58	1	2	EP->WA	0.8739

**Notes.**

EP Eastern Pacific WP Western Pacific WA Western Atlantic Ma millions of years ago BBM Bayesian Binary Method HPD Highest Posterior Density

The pygmy octopuses (*Paroctopus*) species that are distributed in both sides of the Isthmus of Panama formed a highly-supported monophyletic clade (clade 3, PP = 1). These *Paroctopus* species diverged from the most recent common at 17.44 Ma (8.99, 30.31 95% HPD).

The long-armed mimic octopuses *Macrotritopus defilippi* from the Western Atlantic and Octopodidade sp. (White V, as referred by Norman, 2000) from the Western Pacific are sister taxa (PP = 1) and are estimated to have diverged 7.83 Ma (2.82, 15.88 95% HPD), as shown in clade 4.

The transisthmian sister species complex in clade 5 form a well-supported monophyletic group (PP = 1) and were estimated to have had a common ancestor 8.03 Ma (4.27, 13.58 95% HPD) (PP = 1). In this clade, *O. insularis, O. maya* and *O. hummelincki* from WA are the sister taxa to *O. mimus* and *O. hubbsorum* from EP and seem to have diverged after a vicariance event, indicated by the RASP analysis.

### Biogeographical analysis

Reconstruct Ancestral State in Phylogenies (RASP) showed that the most recent common ancestors of the TSSP/TSSC originated in the Pacific Ocean (Table 2). Clades 1, 3 and 5 had high probabilities of an ancestral distribution in the East Pacific (PP = 0.88, 0.87, 0.87, respectively). Clades 2 and 4 were estimated to have originated from ancestors distributed in the Western Pacific Ocean (PP = 0.37 and 0.88, respectively). The dispersal routes for each TSSP/TSSC clade estimated by Bayesian Binary Method are shown in the Table 2.

The biogeographical analysis revealed five vicariant and ten dispersal events on the well-supported nodes of five transisthmian species pair/complex during the early Miocene to early Pliocene (Table 2). The time-event curve (TEC) analysis shows an increase of vicariant and dispersal events after the middle Miocene (∼15 Ma), with a peak in the Early Pliocene (∼5 Ma).

## Discussion

Bayesian phylogenetic inference revealed transisthmian sister species pair/complexes in three genera of octopod species and low probabilities in two genera. These species diverged between 18 and 5 Ma, before the closure of the Isthmus of Panama suggested by the Late Pliocene model (3 Ma). The ancestral area reconstruction analysis showed vicariant events on each node of TSSP/TSSC and an increase of dispersal and vicariance during the process of isthmus formation, which indicates an influence of the emergence of the geological barrier (Isthmus of Panama) on the divergence processes among octopod species. In addition, the RASP results suggest that the most recent common ancestor of the five clades occupied the Pacific Ocean, and the most probable route of dispersal before the closure of the IP is from Pacific to Atlantic.

The divergence times estimated in the present study revealed that octopods TSSP/TSSC from Atlantic and Pacific diverged between the Middle Miocene and Early Pliocene (mean range = 5–18 Ma). This suggests that after 15 Ma (age of the isthmus final closure in the Miocene model), there was likely sufficient connectivity between Caribbean and Pacific oceans to maintain dispersal for some octopus species between these locations up until as recently as the Early Pliocene.

The emergence of the Isthmus of Panama was a long process that caused profound but gradual changes in a range of oceanographic conditions, including temperature, salinity, circulation and productivity (O’Dea et al., 2016). The seaway is understood to have been shallowing by the Middle Miocene, decreasing in depth from over 2,000 m to less than 1,000 m deep (Coates, 1997). Reorganisations of ocean circulation from eastward-flowing to westward-flowing occurred during the Late Oligocene increased productivity within the Caribbean during the Early Miocene (Bartoli et al., 2005; Schneider & Schmittner, 2006). Temperature and salinity began to increase approximately 7 Ma (Collins, 1996). Around 4 Ma, the narrowing of the seaway began to extinguish Caribbean upwelling and the primary productivity of this region dropped dramatically, while it increased in the Eastern Pacific (Pennington et al., 2006; Lessios, 2008; Coates & Stallard, 2013; Leigh, O’Dea & Vermeij, 2013).

Considering that the oceanographic changes were so strong as to affect the global climate (Lear, Rosenthal & Wright, 2003), we suggest that octopods TSSP/TSSC probably diverged as a consequence of these physical and environmental barriers, even before the complete uplift of the IP 3 Ma suggested by the Late Pliocene model. A revision carried out by Lessios (2008) indicated that 73 from 115 geminate clades, including echinoids, crustaceans, fishes and molluscs, split earlier than the final closure of the IP. A similar result was verified by O’Dea et al. (2016), who used 38 comparisons based on fossil-calibrated phylogenies and revealed that 26 (68%) produced estimates of separation that occurred more recently than 12 Ma. Marko (2002), studying divergences among six pairs of geminates in the Arcidae bivalves, also reported that isolation of geminate species did not necessarily occur in the latest stages of closure of the Central American Seaway.

The soft-bodied cephalopods are poorly represented in the fossil record, which makes estimating divergence times of octopuses challenging (Strugnell et al., 2006). However, divergence times of non-calibrated nodes generated in the present study are consistent with previous studies. Using two biogeographical calibrations (LGM and uplift of the IP), Gleadall (2013) estimated that *Enteroctopus* and *Muusoctopus* lineages separated 22 +/ − 2.2 Ma, which is in accordance with this study (22 Ma, 10-40 95% HPD in this study). The age of separation between the *M. januari* and *M. yaquinae* clades (11 Ma, 5–21 95% HPD) is also in accordance with Gleadall’s (2013) results (13.4 Ma). Furthermore, Amor et al. (2014), using the rate of evolution for COI in cephalopods, estimated 19.0–40.9 Ma for the segregation between the *O. vulgaris* and *O. mimus* groups, which is in accordance with the results of this study (29 Ma, 18–43 95% HPD).

According to the reconstruction ancestral area analysis, the most recent common ancestors of all octopod TSSP/TSSC originated from the Pacific Ocean. The shift in the ocean circulation flow from east–west to west–east would carry paralarvae of many species from the Pacific to the Atlantic before the final closure of the IP (Bartoli et al., 2005; Schneider & Schmittner, 2006). Several studies have proposed that many Caribbean species of bivalve, gastropod and fish also derived from Pacific ancestors (Bermingham, Shawn McCafferty & Martin, 1997; Meyer, 2003; Leigh, O’Dea & Vermeij, 2013; LaBella et al., 2017). Additionally, Leigh, O’Dea & Vermeij (2013) pointed out that fossil evidence indicates a Pacific origin for six gastropod species that occupied both sides of tropical America and have become extinct in the Atlantic.

The earliest divergence of all octopods TSSP/TSSC investigated in this study occurred between the pygmy octopuses *P. digueti* from Eastern Pacific and *P. mercatoris*/*P.* cf. *joubini* from the Western Atlantic 17.44 Ma (8.99, 30.31 95% HPD, clade 3). Lower fecundity rates (20–320 eggs and benthic hatchling) (Forsythe & Toll, 1991) combined with small adult size (body weight from 20 g to 85 g) (Norman, Hochberg & Finn, 2014) may have reduced the ability of these species to maintain dispersal after the first environmental changes resulting from the formation of the IP.

The divergence among the deep-sea species *M. januari* and *M. yaquinae* group was around 11 Ma (5.08, 20.77 95% HPD, clade 1). This split was probably influenced by the shoaling of the IP, which shut off deep water connection between 12 and 9.2 Ma (Coates et al., 1992; Lear, Rosenthal & Wright, 2003). The deep divergences among transisthmian species can also be explained by the extinction of geminates as a consequence of the uplift of the isthmus (Leigh, O’Dea & Vermeij, 2013; Lessios, 1998). Marko (2002) affirms that molluscan transisthmian taxa from the EP and the WA may be rare due to successive events of extinction caused by the seaway closure, in which at least 70% of some molluscan subgeneric groups were lost.

The clade that included *Macrotritopus defilippi* from the Western Atlantic and Octopodidae sp. (White V) (Norman, 2000) from the Western Pacific shared a MRCA up to 8 Ma (2.82, 15.88 95% HPD, clade 4). These sand-dwelling species have similar morphological and complex behavioural characteristics, such as mimicry and ‘flatfish swimming’ (octopus that mimicked the shape, swimming actions, speed, duration and the colouration of swimming flounders) (Hanlon, Conroy & Forsythe, 2008). They also appear to have evolved from a sand-dwelling common ancestor with extremely long arms (Huffard et al., 2010) and with small and planktonic eggs at the Pacific Ocean. However, these relationships and routes of dispersal may be biased because the *M. defilippi* from the Mediterranean Sea (locality of the species holotype) was not available for this analysis.

The most recent divergence in our analysis is the nocturnal *Callistoctopus* species from the Western Pacific and Western Atlantic at 5 Ma (2.21, 10.96 95% HPD, clade 2). *Callistoctopus ornatus* occupies a broad area of the Indian and Western and Central Pacific Oceans, while *C. macropus* occurs in the Mediterranean Sea and Eastern Atlantic Ocean (Norman, Hochberg & Finn, 2014; Voss, 1981). The nocturnal *Callistoctopus* sp. from the Brazilian coast is morphologically, genetically and behaviourally related to *C. ornatus* and *C. macropus* (Leite TS, Lima FD, pers. comm., 2018). Additionally, all the species in clade 2 have small and planktonic hatchlings, which may have facilitated their dispersal and diversification across the seas (Boyle & Rodhouse, 2005; Norman, Hochberg & Finn, 2014). The BBM analysis pointed to the WP–WA as more plausible dispersal route of the nocturnal species, suggesting that the channel before the final closure of the IP was the likely pathway. However, the posterior probability is low (PP BBM route = 0.37), which suggests that alternative routes of colonisation, such as from Indo-Pacific to Eastern Atlantic, were also likely to have been taken. Schneider & Schmittner (2006) pointed out a connection between the tropical gateways of Panama and Indonesia in the way that reduced outflow of upper Pacific Ocean waters via the Panama seaway into the Atlantic is compensated by increased flow towards the Indonesian Archipelago. Given that the divergence of these species occurred shortly before the total closure of the isthmus, this interflow may explain the dispersal of species from the West to East Pacific and, subsequently to the West Atlantic via the Panama Seaway.

Clade 5 includes *Octopus* species with remarkable evolutionary success in term of diversification, distribution and abundance on both sides of the Americas. This TSSC groups includes *O. maya*, *O. hummelincki* and *O. insularis* from the WA and *O. mimus* and *O. hubbsorum* from the EP, which have different reproductive strategies and shared a common ancestor 8 Ma (4.27, 13.58 95% HPD). The species *O. mimus* and *O. hubbsorum* are closely related and may represent a single species (Pliego-Cárdenas et al., 2014). They are the putative transisthmian sister taxa of *O. insularis*, because they also share similar morphological characteristics (medium/large muscular species, ocellus absent, white spots on dorsal mantle, skin texture of irregular patches and a groove system), habitat preferences (reefs and rocky bottoms in shallow waters) and life history (small and planktonic eggs) (Leite et al., 2008; Leite et al., 2009; Norman, Hochberg & Finn, 2014).

Prior to the divergence of the species in clade 5, speciation processes appear to have occurred within the Eastern Pacific around 19 Ma (10–29 95% HPD). These processes led to a clade containing two ocellated species with different reproductive traits (*O. bimaculoides* and *O. bimaculatus*, holobenthic and pelagobenthic hatchlings, respectively) in EP and another clade containing the endemic ocellated species from the Galapagos, *O. oculifer* (holobenthic mode), and the species of clade 5. The divergence between *O. oculifer* and clade 5 (12 Ma, 7–20 95% HPD) occurred slightly after the formation of seamounts in Galápagos at 14.5 Ma (Werner et al., 1999), suggesting that this species successfully settled around the rising island and became endemic due to its low dispersive ability.

Even though the divergence of *O. oculifer* was not precipitated by a vicariance event related to the formation of IP, the subsequent evolutionary processes were and seem to have generated a species with very similar characteristics in WA, *O. maya*. They share important biological features, such as large eggs and benthic hatchlings (Arreguín-Sánchez, Solís & González De La Rosa, 2000), which means they probably inherited these traits from a common ancestor. Although some studies have pointed *O. bimaculatus* as the transisthmian sister taxon of *O. maya* (Voight, 1988; Juárez, Rosas & Arenta-Ortiz, 2012; Nesis, 2003; Allcock, Lindgren & Strugnell, 2015), this study suggests that *O. oculifer* is the sister transisthmian species of *O. maya*, since they also share morphological, behavioural and ecological similarities.

## Conclusions

The long geological history of the Isthmus of Panama had an immense impact on the speciation processes of marine biota in the Atlantic and Pacific Oceans (Coates et al., 1992; Bartoli et al., 2005; O’Dea et al., 2007; Coates & Stallard, 2013; O’Dea et al., 2016). The divergence processes among octopods TSSP/TSSC probably occurred up to 5 Ma (2.21 Ma, lower bound of the 95% HPD interval), a long time after the final closure of the IP proposed by the Miocene model, 15 Ma. Considering the influence of the extreme environmental changes during this geological event in the speciation processes, this study indicates that the divergence times of the octopod species are according to the classic Pliocene model.

The results obtained in this phylogenetic reconstruction suggest that it is more probable that the differences among the lineages of octopod transisthmian species arose due to the allopatric speciation process caused by the uplift of Isthmus of Panama than that they arose due to an independent process of evolutionary convergence.

##  Supplemental Information

10.7717/peerj.8691/supp-1 Figure S1 Bayesian phylogenetic tree including all cephalopods species used in this study The bars on the nodes represent the 95% Highest Posterior Density intervals. The 95% HPD of calibrated nodes with three fossils information are shown in orange bars. The asterisk represents the biogeographical calibration. The mean ages of clades divergence are placed below each node (Ma).

10.7717/peerj.8691/supp-2 Table S1 Details of the specimens for COI, 16S rDNA, Rhodopsin and EF1-alpha genes used to construct the final Bayesian phylogenetic tree in this study GB = GenBank accession number, MORG = Museu Oceanográfico do Rio Grande, CTR = Coleção de tecidos de invertebrados da UFRN.

10.7717/peerj.8691/supp-3 Supplemental Information 3 Sequences of 16S mitochondrial genes

10.7717/peerj.8691/supp-4 Supplemental Information 4 Sequences of COI mitochondrial genes

10.7717/peerj.8691/supp-5 Supplemental Information 5 Sequences of EF-1 alpha nuclear genes

10.7717/peerj.8691/supp-6 Supplemental Information 6 Sequences of rhodopsin nuclear genes

## Additional Information and Declarations

### Field Study Permissions

The following information was supplied relating to field study approvals (i.e., approving body and any reference numbers): Field experiments were approved by the Instituto Chico Mendes de Conservação da Biodiversidade, ICMBIO (107065 and 304841).

### Data Availability

The following information was supplied regarding data availability: The sequences are available in GenBank (accessions are listed in Table S1).

## References

[ref-1] Allcock AL, Lindgren A, Strugnell JM (2015). The contribution of molecular data to our understanding of cephalopod evolution and systematics: a review. Journal of Natural History.

[ref-2] Allcock AL, Strugnell J, Johnson MP (2008). How useful are the recommended counts and indices in the systematics of the Octopodidae (Mollusca: Cephalopoda). Biological Journal of the Linnean Society.

[ref-3] Amor MD, Norman MD, Cameron HE, Strugnell JM (2014). Allopatric speciation within a cryptic species complex of Australasian octopuses. PLOS ONE.

[ref-4] Arreguín-Sánchez F, Solís MJ, González De La Rosa ME (2000). Population dynamics and stock assessment for *Octopus maya* (Cephalopoda: Octopodidae) fishery in the Campeche Bank, Gulf of Mexico. Revista de Biologia Tropical.

[ref-5] Bacon CD, Mora A, Wagner WL, Jaramillo CA (2013). Testing geological models of evolution of the Isthmus of Panama in a phylogenetic framework. Botanical Journal of the Linnean Society.

[ref-6] Bacon CD, Silvestro D, Jaramillo C, Smith BT, Chakrabarty P, Antonelli A (2015). Biological evidence supports an early and complex emergence of the Isthmus of Panama. Proceedings of the National Academy of Sciences of the United States of America.

[ref-7] Bartoli G, Sarnthein M, Weinelt M, Erlenkeuser H, Lea DW (2005). Final closure of Panama and the onset of northern hemisphere glaciation. Earth and Planetary Science Letters.

[ref-8] Bermingham E, Lessios HA (1993). Rate variation of protein and mitochondrial DNA evolution as revealed by sea urchins separated by the Isthmus of Panama. Proceedings of the National Academy of Sciences of the United States of America.

[ref-9] Bermingham E, Shawn McCafferty S, Martin AP, Kocher TD, Stepien CA (1997). Fish biogeography and molecular clocks: perspectives from the Panamanian Isthmus. Molecular systematics of fishes.

[ref-10] Boyle P, Rodhouse PG (2005). Cephalopods: ecology and fisheries.

[ref-11] Castresana J (2000). Selection of conserved blocks from multiple alignments for their use in phylogenetic analysis. Molecular Biology and Evolution.

[ref-12] Coates AG, Coates AG (1997). The forging of Central America. Central America: a natural and cultural history.

[ref-13] Coates AG, Collins LS, Aubry MP, Berggren WA (2004). The geology of the Darien, Panama, and the late Miocene-Pliocene collision of the Panama arc with Northwestern South America. Geological Society of America Bulletin.

[ref-14] Coates AG, Jackson JBC, Collins LS, Cronin TM, Dowsett HJ, Bybell LM, Jung P, Obando JA (1992). Closure of the Isthmus of Panama: the near-shore marine record of Costa Rica and Western Panama. Geological Society of America Bulletin.

[ref-15] Coates AG, Stallard RF (2013). How old is the isthmus of Panama?. Bulletin of Marine Science.

[ref-16] Collins T, Jackson JBC, Budd AF, Coates AG (1996). Molecular comparisons of transisthmian species pairs: rates and patterns of evolution. Evolution and environment in tropical America.

[ref-17] Cowman PF, Bellwood DR (2013). Vicariance across major marine biogeographic barriers: temporal concordance and the relative intensity of hard versus soft barriers. Proceedings of the Royal Society B: Biological Sciences.

[ref-18] Drummond AJ, Suchard MA, Xie D, Rambaut A (2012). Bayesian phylogenetics with BEAUti and the BEAST 1.7. Molecular Biology and Evolution.

[ref-19] Fisher JC, Riou B (2002). Vampyronassa rhodanica nov. gen. nov. sp., vampyromorpha (Cephalopoda, Coleoidea) du Callovien inférieur de la Voulte-sur-Rhône (Ardéche, France). Annales de Paléontologie.

[ref-20] Folmer O, Black M, Hoeh W, Lutz R, Vrijenhoek R (1994). DNA primers for amplification of mitochondrial cytochrome c oxidase subunit I from diverse metazoan invertebrates. Molecular Marine Biology and Biotechnology.

[ref-21] Forsythe JW, Toll RB (1991). Clarification of the Western Atlantic Ocean pygmy octopus complex: the identity and life history of *Octopus joubini* (Cephalopoda: Octopodinae). Bulletin of Marine Science.

[ref-22] Fuchs D, Bracchi G, Weis R (2009). New Octopods (Cephalopoda: Coleoidea) from the Late Cretaceous (Upper Cenomanian) of Hâkel Hândjoula, Lebanon. Palaeontology.

[ref-23] Galván-Quesada S, Doadrio I, Alda F, Perdices A, Reina RG, Varela MG, Hernández N, Mendoza AC, Bermingham E (2016). Molecular phylogeny and biogeography of the amphidromous fish genus dormitator gill 1861 (Teleostei: Eleotridae). PLOS ONE.

[ref-24] Gleadall IG (2013). A molecular sequence proxy for *Muusoctopus januarii* and calibration of recent divergence among a group of mesobenthic octopuses. Journal of Experimental Marine Biology and Ecology.

[ref-25] Graham JB (1971). Temperature tolerances of some closely related tropical Atlantic and Pacific fish species. Science.

[ref-26] Hanlon RT, Conroy LA, Forsythe JW (2008). Mimicry and foraging behaviour of two tropical sand-flat octopus species off North Sulawesi, Indonesia. Biological Journal of the Linnean Society.

[ref-27] Haug GH, Tiedemann R (1998). Effect of the formation of the Isthmus of Panama on Atlantic Ocean thermohaline circulation. Letters to Nature.

[ref-28] Hoorn C, Flantua S (2015). An early start for the Panama land bridge. Science.

[ref-29] Huffard CL, Saarman N, Hamilton H, Simison WB (2010). The evolution of conspicuous facultative mimicry in octopuses: an example of secondary adaptation?. Biological Journal of the Linnean Society.

[ref-30] Iglesias J, Sanchez F, Bersano J, Carrasco J, Dhont J, Fuentes L, Linares F, Munoz J, Okumura S, Roo J (2007). Rearing of Octopus *vulgaris* paralarvae: present status, bottlenecks and trends. Aquaculture.

[ref-31] Juárez O, Rosas C, Arenta-Ortiz M (2012). Phylogenetic relationships of *Octopus maya* revealed by mtDNA sequences. Ciencias Marinas.

[ref-32] Kearse M, Moir R, Wilson A, Stones-Havas S, Cheung M, Sturrock S, Buxton S, Cooper A, Markowitz S, Duran C, Thierer T, Ashton B, Meintjes P, Drummond A (2012). Geneious basic: an integrated and extendable desktop software platform for the organization and analysis of sequence data. Bioinformatics.

[ref-33] Keigwin L (1982). Isotopic paleoceanography of the Caribbean. Science.

[ref-34] Knowlton N (2000). Molecular genetic analyses of species boundaries in the sea. Hydrobiologia.

[ref-35] Knowlton N, Weigt LA (1998). New dates and new rates for divergence across the Isthmus of Panama. Proceedings of the Royal Society of London B.

[ref-36] Knowlton N, Weigt LA, Solórzano LA, Mills DK, Bermingham E (1993). Divergence in protein, mitochondrial DNA, and reproductive compatibility across the Isthmus of Panama. Science.

[ref-37] Kobayashi T (1954). A new Palaeogene paracenoceratoid from Southern Kyushu in Japan. Japanese Journal of Geology and Geography.

[ref-38] Kröger B, Vinther J, Fuchs D (2011). Cephalopod origin and evolution: a congruent picture emerging from fossils, development and molecules. Bioessays.

[ref-39] LaBella AL, Van Dover CL, Jollivet D, Cunningham CW (2017). Gene flow between Atlantic and Pacific Ocean basins in three lineages of deep-sea clams (Bivalvia: Vesicomyidae: Pliocardiinae) and subsequent limited gene flow within the Atlantic. Deep-Sea Research II.

[ref-40] Lear CH, Rosenthal Y, Wright JD (2003). The closing of a seaway: ocean water masses and global climate change. Earth and Planetary Science Letters.

[ref-41] Leigh EG, O’Dea A, Vermeij GJ (2013). Historical biogeography of the Isthmus of Panama. Biological Reviews.

[ref-42] Leite TS, Haimovici M, Mather J, Oliveira JEL (2009). Habitat, distribution, and abundance of the commercial octopus (*Octopus insularis*) in a tropical oceanic island, Brazil: information for management of an artisanal fishery inside a marine protected area. Fisheries Research.

[ref-43] Leite TS, Haimovici M, Molina W, Warnke K (2008). Morphological and genetic description of *Octopus insularis*, a new cryptic species in the *Octopus vulgaris* complex (Cephalopoda: Octopodidae) from the tropical Southwestern Atlantic. Journal of Molluscan Studies.

[ref-44] Lessios HA (1981). Divergence in allopatry: molecular and morphological differentiation between sea urchins separated by the Isthmus of Panama. Evolution.

[ref-45] Lessios H, Howard DJ, Berlocher SH (1998). The first stage of speciation as seen in organism separated by the Isthmus of Panama. Species and speciation.

[ref-46] Lessios HA (2008). The great American schism: divergence of marine organisms after the rise of the Central American Isthmus. Annual Review of Ecology, Evolution, and Systematics.

[ref-47] Lessios HA, Weinberg JR (1994). Genetic and morphological divergence among Morphotypes of the isopod excirolana on the two sides of the Isthmus of Panama. Society.

[ref-48] Mangold K, Boyle PR (1987). Reproduction. Cephalopod life cycles II—comparative reviews.

[ref-49] Marek C (2015). Dissertation The emergence of the Isthmus of Panama—a biological perspective. Ph.D. Thesis.

[ref-50] Marko PB (2002). Fossil calibration of molecular clocks and the divergence times of geminate species pairs separated by the Isthmus of Panama. Molecular Biology and Evology.

[ref-51] Meyer CP (2003). Molecular systematics of cowries (Gastropoda: Cypraeidae) and diversification patterns in the tropics. Biological Journal of the Linnean Society.

[ref-52] Montes C, Cardona A, Jaramillo C, Pardo A, Silva JC, Valencia V, Ayala C, Pérez-Angel LC, Rodriguez-Parra LA, Ramirez V, Niño H (2015). Middle Miocene closure of the Central American Seaway. Science.

[ref-53] Montes C, Cardona A, McFadden R, Morón SE, Silva CA, Restrepo-Moreno S, Ramírez DA, Hoyos N, Wilson J, Farris D, Bayona GA, Jaramillo CA, Valencia V, Bryan J, Flores JA (2012). Evidence for middle Eocene and younger land emergence in Central Panama: implications for Isthmus closure. Geological Society of America Bulletin.

[ref-54] Nesis KN (2003). Distribution of recent Cephalopoda and implications for Plio-Pleistocene events. Berliner Paläobiologische Abhandlungen.

[ref-55] Norman M (2000). Cephalopods—a world guide.

[ref-56] Norman MD, Hochberg FG, Finn JK, Jereb P, Roper CFE, Norman MD, Finn JK (2014). Octopus and vampire squids. Cephalopods of the World. An annotated and illustrated catalogue of cephalopod species known to date.

[ref-57] O’Dea A, Jackson JBC, Fortunato H, Smith JT, D’Croz L, Johnson KG, Todd JA (2007). Environmental change preceded Caribbean extinction by 2 million years. Proceedings of the National Academy of Sciences of the United States of America.

[ref-58] O’Dea AO, Lessios HA, Coates AG, Eytan RI, Restrepo-moreno SA, Cione AL, Collins LS, De Queiroz A, Farris DW, Norris RD, Stallard RF, Woodburne MO, Aguilera O, Aubry M, Berggren WA, Budd AF, Cozzuol MA, Coppard SE, Duque-caro H, Finnegan S, Gasparini GM, Grossman EL, Johnson KG, Keigwin LD, Knowlton N, Leigh EG, Leonard-pingel JS, Marko PB, Pyenson ND, Rachello-dolmen PG, Soibelzon E, Soibelzon L, Todd JA, Vermeij GJ, Jackson JBC (2016). Formation of the Isthmus of Panama. Science Advances.

[ref-59] Palumbi SR (1994). Genetic divergence, reproductive isolation, and marine speciation. Annual Review of Ecology and Systematics.

[ref-60] Palumbi SR, Hillis DM, Moritz C, Mable BK (1996). Nucleic acids II: the polymerase chain reaction. Molecular systematics.

[ref-61] Parham JF, Donoghue PCJ, Bell CJ, Calway TD, Head JJ, Holroyd PA, Inoue JG, Irmis RB, Joyce WG, Ksepka DT, Patané JSL, Smith ND, Tarver JE, Van Tuinen M, Yang Z, Angielczyk KD, Greenwood JM, Hipsley CA, Jacobs L, Makovicky PJ, Müller J, Smith KT, Theodor JM, Warnock RCM, Benton MJ (2012). Best practices for justifying fossil calibrations. Systematic Biology.

[ref-62] Pennington JT, Mahoney KL, Kuwahara VS, Kolber DD, Calienes R, Chavez FP (2006). Primary production in the eastern tropical Pacific: a review. Progress in Oceanography.

[ref-63] Pliego-Cárdenas R, Hochberg FG, De León FJG, Barriga-Sosa IDLA (2014). Close genetic relationships between two American octopuses: octopus hubbsorum Berry, 1953, and *Octopus mimus* Gould, 1852. Journal of Shellfish Research.

[ref-64] Posada D (2008). jModelTest: phylogenetic model averaging. Molecular Biology and Evolution.

[ref-65] Rambaut A, Suchard MA, Xie D, Drummond AJ (2014). http://tree.bio.ed.ac.uk/software/tracer/.

[ref-66] Robertson DR, Collin R (2015). Inter- and intra-specific variation in egg size among reef fishes across the Isthmus of Panama. Frontiers in Ecology and Evolution.

[ref-67] Sanchez G, Setiamarga DHE, Tuanapaya S, Tongtherm K, Winkelmann IE, Schmidbaur H, Umino T, Albertin C, Allcock L, Perales-Raya C, Gleadall I, Strugnell JM, Simakov O, Nabhitabhata J (2018). Genus-level phylogeny of cephalopods using molecular markers: current status and problematic areas. PeerJ.

[ref-68] Schneider B, Schmittner A (2006). Simulating the impact of the Panamanian seaway closure on ocean circulation, marine productivity and nutrient cycling. Earth and Planetary Science Letters.

[ref-69] Stone R (2014). Battle for the Americas. Science.

[ref-70] Strugnell J, Jackson J, Drummond AJ, Cooper A (2006). Divergence time estimates for major cephalopod groups: evidence from multiple genes. Cladistics.

[ref-71] Strugnell JM, Rogers AD, Prodöhl PA, Collins MA, Allcock AL (2008). The thermohaline expressway: the Southern Ocean as a centre of origin for deep-sea octopuses. Cladistics.

[ref-72] Tamura K, Stecher G, Peterson D, Filipski A, Kumar S (2013). MEGA6: molecular evolutionary genetics analysis version 6.0. Molecular Biology and Evolution.

[ref-73] Voight JR (1988). Trans-Panamanian geminate octopods (Mollusca:Octopoda). Malacologia.

[ref-74] Voss GL (1981). A redescription of Octopus ornatus Gould, 1852 (Octopoda: Cephalopoda) and the status of Callistoctopus Taki, 1964. Proceedings of the Biological Society of Washington.

[ref-75] Werner R, Hoernle K, Van Den Bogaard P, Ranero C, Von HueneR, Korich D (1999). Drowned 14-m.y.-old Galápagos archipelago off the coast of Costa Rica: implications for tectonic and evolutionary models. Geology.

[ref-76] Yu Y, Harris AJ, Blair C, He X (2015). RASP (Reconstruct Ancestral State in Phylogenies): a tool for historical biogeography. Molecular Phylogenetics and Evolution.

