# Supplementary figures and images for: A biogeographic framework of octopod species diversification: the role of the Isthmus of Panama
Source: PeerJ. 2020 Mar 27;8:e8691. doi: 10.7717/peerj.8691 (PMC7104719; doi:10.7717/peerj.8691)

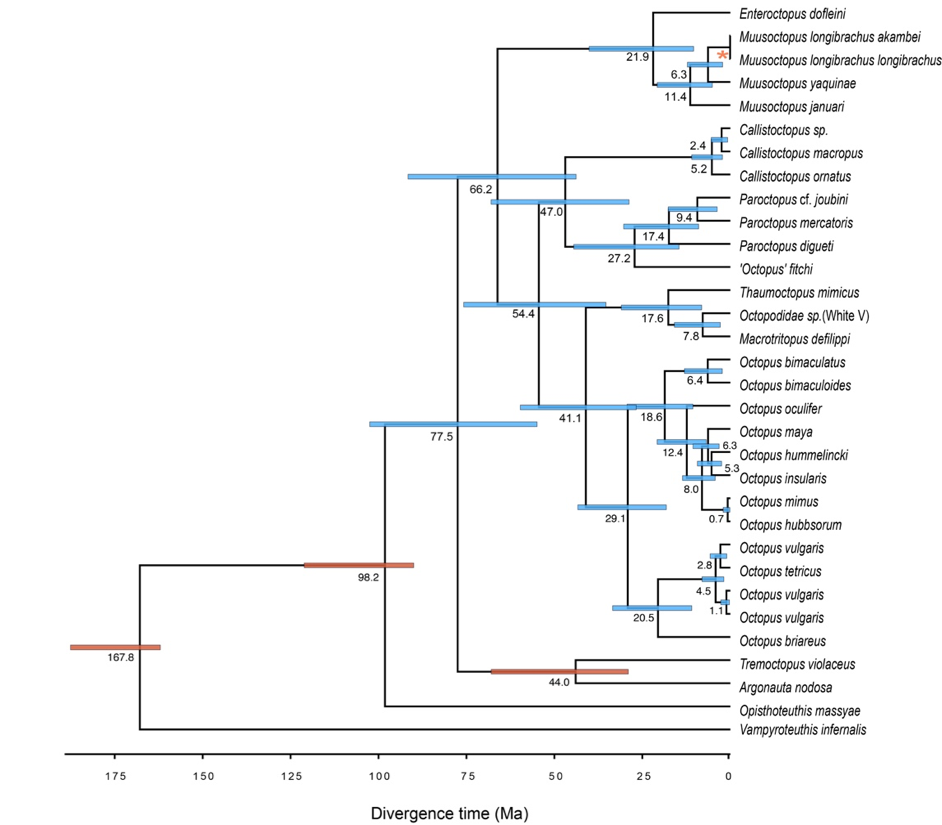

Supplement: Figure S1 — The bars on the nodes represent the 95% Highest Posterior Density intervals. The 95% HPD of calibrated nodes with three fossils information are shown in orange bars. The asterisk represents the biogeographical calibration. The mean ages of clades divergence are placed below each node (Ma). [file peerj-08-8691-s001.png]
