# Supplementary material for: A biogeographic framework of octopod species diversification: the role of the Isthmus of Panama
Source: PeerJ. 2020 Mar 27;8:e8691. doi: 10.7717/peerj.8691 (PMC7104719; doi:10.7717/peerj.8691)
Supplement: Table S1 — GB = GenBank accession number, MORG = Museu Oceanográfico do Rio Grande, CTR = Coleção de tecidos de invertebrados da UFRN. [file peerj-08-8691-s002.docx]

**Table S1** **Details of the specimens for COI, 16S rDNA, Rhodopsin and EF1-alpha genes used to construct the final Bayesian phylogenetic tree in this study.** GB = GenBank accession number, MORG = Museu Oceanográfico do Rio Grande, CTR = Coleção de tecidos de invertebrados da UFRN.

| **Species** | **Local** | **COI** | | **16S** | | **RHODOPSIN** | | **EF1-ALPHA** | | **Voucher number** |
| --- | --- | --- | --- | --- | --- | --- | --- | --- | --- | --- |
|  |  | **GB** | **Reference** | **GB** | **Reference** | **GB** | **Reference** | **GB** | **Reference** |  |
| *Octopus bimaculatus* | EP | KT335828 | Pliego-Cardenas et al. 2014 | KT335834 | Pliego-Cardenas et al. 2014 | KT335846 | Pliego-Cardenas et al. 2014 | - |  |  |
| *Octopus bimaculoides* | EP | KF225006 | Pliego-Cardenas et al. 2014 | KF373765 | Pliego-Cardenas et al. 2014 | KT335847 | Pliego-Cardenas et al. 2014 | JF927854 | Kroger et al. 2011 |  |
| *Octopus briareus* | WA | MN933636 | This study | - | - | - | - | - | - | CRT 4854 |
| *Octopus hubbsorum* | EP | KF225002 | Pliego-Cardenas et al. 2014 | KF373764 | Pliego-Cardenas et al. 2014 | - | - | - | - |  |
| *Octopus hummelincki* | WA | MN933640, MN933641 | This study | MN508071, MN508071 | This study | MN946386,MN946387 | This study | MN946371 | This study | MORG 52760, CRT 4858 |
| *Octopus insularis* | WA | MN933642- MN933644 | This study | MN508072- MN508074 | This study | MN946388-MN946390 | This study | MN946376- MN946378 | This study | MORG 49524, CRT 4860, CRT 4861 |
| *Octopus maya* | WA | MN933647, MN933648 | This study | MN508077, MN508078 | This study | MN946392,MN946393 | This study | MN946372, MN946373 | This study | CRT 4865, CRT 4866 |
| *Paroctopus mercatoris* | WA | GQ900743 | Huffard et al. 2010 | GQ900704 | Huffard et al. 2010 | - | - | - | - |  |
| *Octopus mimus* | EP | KT335830 | Pliego-Cardenas et al. 2014 | KT362707 | Pliego-Cardenas et al. 2014 (unpubl.) | KT335848 | Pliego-Cardenas et al. 2014 | - | - |  |
| *Octopus oculifer* | EP | KT335831 | Pliego-Cardenas et al. 2014 | KT335837 | Pliego-Cardenas et al. 2014 | KT335849 | Pliego-Cardenas et al. 2014 | - | - |  |
| *Octopus tetricus* | WP | KJ605260 | Amor et al. 2015 | KJ605236 | Amor et al. 2015 | - | - | AY651882 |  |  |
| *Octopus vulgaris* | EA | HQ908433 | Keskin and Atar 2011 | AJ252778 | Hudelot *unpublished* | EF016312 | Allcock et al. 2006 | AY651883 |  |  |
| *Octopus vulgaris* | WA | MN933649- MN933651 | This study | MN508080- MN508082 | This study | MN946394- MN946396 | This study | MN946374, MN946375 | This study | CRT 4868-CRT 4870 |
| *Paroctopus digueti* | EP | KT335833 | Pliego-Cardenas et al. 2014 | KT335839 | Pliego-Cardenas et al. 2014 | KT335851 | Pliego-Cardenas et al. 2014 | - | - |  |
| *Octopus fitchi* | EP | KT335832 | Pliego-Cardenas et al. 2014 | KT335838 | Pliego-Cardenas et al. 2014 | KT335850 | Pliego-Cardenas et al. 2014 | - | - |  |
| *Paroctopus* cf. *joubini* | WA | MN933645, MN933646 | This study | MN508075- MN508076 | This study | MN946391 | This study | - | - | MORG 52754 |
| *Callistoctopus* sp*.* | WA | MN933632 | This study | MN508063 | This study | - | - | - | - | MORG 51424 |
| *Callistoctopus macropus* | EA | MN933633- MN933635 | This study | MN508064- MN508066 | This study | MN946381- MN946383 | This study | - | - | MORG 51380, CRT 4851, CRT 4852 |
| *Callistoctopus ornatus* | WP | HM104257 | Strugnell et al. 2013 | AB191114 | Takumiya et al. 2004 | AY616926 | Strugnell et al. 2005 | - | - |  |
| *Macrotritopus defilippi* | WA | MN933637- MN933639 | This study | MN508067- MN508069 | This study | MN946384, MN946385 | This study | - | - | MORG 52748, CRT 4856 |
| *Octopodidae sp.* (White V) | WP | GQ900737 | Huffard et al. 2010 | GQ900722 | Huffard et al. 2010 | - | - | - | - |  |
| *Thaumoctopus mimicus* | WP | GQ900746 | Huffard et al. 2010 | GQ900725 | Huffard et al. 2010 | - | - | - | - |  |
| *Enteroctopus dofleini* | EP | GU802397 | Layton et al. 2014 | AB191107 | Takumiya et al. 2004 | AY545174 | Strugnell et al. 2004 | - | - |  |
| *Muusoctopus longibrachus akambei* | EA | HM572177 | Strugnell et al. 2011 | HM572157 | Strugnell et al. 2011 | HM572220 | Strugnell et al. 2011 | - | - |  |
| *Muusoctopus januari* | WA | EF016335 | Allcock et al. 2006 | EF016344 | Allcock et al. 2006 | HM572223 | Strugnell et al. 2011 | - | - |  |
| *Muusoctopus longibrachus longibrachus* | EP | HM572169 | Strugnell et al. 2011 | HM572166 | Strugnell et al. 2011 | HM572219 | Strugnell et al. 2011 | - | - |  |
| *Muusoctopus yaquinae* | EP | HM572182 | Strugnell et al. 2011 | FJ603539 | Strugnell et al. 2009 | GQ226017 | Strugnell et al. 2009 | - | - |  |
| *Tremoctopus violaceus* | WA | AF377978 | Carlini et al. 2001 | AJ252767 | Hudelot, *unpublished* | AY545167 | Strugnell et al. 2004 | - | - |  |
| *Argonauta nodosa* | WP | AY557517 | Lindgren et al. 2004 | AY545104 | Strugnell et al. 2004 | AY545166 | Strugnell et al. 2004 | AY651855 | Guzik et al. 2005 |  |
| *Opisthoteuthis massyae* | EA | AY545187 | Strugnel et al. 2004 | AY545103 | Strugnell et al. 2004 | HM104301 | Strugnell et al. 2014 | AY651877 | Guzik et al. 2005 |  |
| *Vampyroteuthis infernalis* | WP | AB385880 | Kaneko et al. 2008 | AY545101 | Strugnell et al. 2004 | AY545163 | Strugnell et al. 2004 | - | - |  |
