# Supplementary material for: A biogeographic framework of octopod species diversification: the role of the Isthmus of Panama
Source: PeerJ. 2020 Mar 27;8:e8691. doi: 10.7717/peerj.8691 (PMC7104719; doi:10.7717/peerj.8691)
Supplement: Supplemental Information 3 [file peerj-08-8691-s003.pdf]

>383
AACAGCTGCGGTATTATAACTGTACTAAGGTAGCATAATAATTTGCCTTGTAAATTAAGGCTAGAATG
AATGGTTTGACGAAAATTAAGCTGTCTCTAATTTATTTGTTAGAAATTAATTTTTATAGTGAAAAAGC
TTGGATAATTTAAAGGGACGAGAAGACCCTATTGAGCTTTAATAATATATTTTGATTGGGGTGATCAA
GGAATAAAACTTCCTTATTAATTGAGTAATAAACCAAAGTTTTTGCTTATAAGATAAGTTACCATAGG
GGTAACAGCGTAATTTTTTTTGAGAGTTCATATTGAAAAAAGAGATTGCGACCTCGATGTTGGATTAA
AATACCTTAAGGTGAAGAGGCTTTATTAGGTGAATCTGTTCGATTTTTAAAATTTT
>384
AACAGCTGCGGTATTATAACTGTACTAAGGTAGCATAATAATTTGCCTTGTAAATTAAGGCTAGAATG
AATGGTTTGACGAAAATTAAGCTGTCTCTAATTTATTTGTTAGAAATTAATTTTTATAGTGAAAAAGC
TTGGATAGTTTAAAGGGACGAGAAGACCCTATTGAGCTTTAATAATATATTTTGATTGGGGGGATCAA
GGAATAAAACTTCCTTATTAATTGAGTAATAAACCAAAGTTTTTGCTTATAAGATAAGTTACCATAGG
GGTAACAGCGTAATTTTTTTTGAGAGTTCATATTGAAAAAAGAGATTGCGACCTCGATGTTGGATTAA
AATACCTTAAGGTGAAGAGGCTTTATTAGGTGAATCTGTTCGATTTTTAAAATTTT
>385
AACAGCTGCGGTATTATAACTGTACTAAGGTAGCATAATAATTTGCCTTGTAAATTAAGGCTAGAATG
AATGGTTTGACGAAAATTAAGCTGTCTCTAATTTATTTGTTAGAAATTAATTTTTATAGTGAAAAAGC
TTGGATAGTTTAAAGGGACGAGAAGACCCTATTGAGCTTTAATAATATATTTTGATTGGGGTGATCAA
GGAATAAAACTTCCTTATTAATTGAGTAATAAACCAAAGTTTTTGCTTATAAGATAAGTTACCATAGG
GGTAACAGCGTAATTTTTTTTGAGAGTTCATATTGAAAAAAGAGATTGCGACCTCGATGTTGGATTAA
AATACCTTAAGGTGAAGAGGCTTTATTAGGTGAATCTGTTCGATTTTTAAAATTTT
>386
AACAGCTGCGGTATTATAACTGTACTAAGGTAGCATAATAATTTGCCTTGTAAATTAAGGCTAGAATG
AATGGTTTGACGAAAATTAAGCTGTCTCTAATTTATTTGTTAGAAATTAATTTTTATAGTGAAAAAGC
TTGGATAGTTTAAAGGGACGAGAAGACCCTATTGAGCTTTAATAATATATTTTGATTGGGGTGATCAA
GGAATAAAACTTCCTTATTAATTGAGTAATAAACCAAAGTTTTTGCTTATAAGATAAGTTACCATAGG
GGTAACAGCGTAATTTTTTTTGAGAGTTCATATTGAAAAAAGAGATTGCGACCTCGATGTTGGATTAA
AATACCTTAAGGTGAAGAGGCTTTATTAGGTGAATCTGTTCGATTTTTAAAATTTT
>bimaculatus
AACAGCTGCGGTATTATAACTGTACTAAGGTAGCATAATAATTTGCTCTATAAATTGGGGCTAGAATG
AATGGTTTGACGAAAATTTAACTGTCTCTATTTTATTTATTAGAAATTAATTTTTATAGTGAGAAAGC
TTAAATTGTTTAAAGGGACGAAAAGACCCTATTGAGCTTTATTAATTTATTTTGGTTGGGGTGATCAA
GGAATAAAACTTCCTTATTTTTGGAAAAATAAACCAAGTTTTTTGCTTAGAAGATAAGTTACCATAGG
GATAACAGCGTAATTTTTTTTGAGAGTTCGTATTGAAAAAAGAGATTGCGACCTCGATGTTGGATTAA
AAAACCTTAAGGTGTAGAGGCTTTGTTAGGTAAATCTGTTCGATTTTTAAAATTTT
>bimaculoides1
AACAGCTGCGGTATTATAACTGTACTAAGGTAGCATAGTAATTTGCTCTATAAATTGGGGCTAGAATG
AATGGTTTGACGAAAATTTAACTGTCTCTATTTTATTTATTAGAAATTAATTTTTATAGTGAAAAAGC
TTAAATTATTTAAAGGGACGAAAAGACCCTATTGAGCTTTATTAATTTATTTTGATTGGGGTGATCAA
GGAATAAAACTTCCTTATTTTTGGAAAAATAAACCAAGTTTTTTGCTTAGAAGATAAGTTACCATAGG
GATAACAGCGTAATTTTTTTTGAGAGTTCATATTGAAAAAGGAGATTGCGACCTCGATGTTGGATTAA
AAAACCTTAAGGTGTAGAGGCTTTATTAGGTAAATCTGTTCGATTTTTAAAATTTT
>bimaculoides2
AACAGCTGCGGTATTATAACTGTACTAAGGTAGCATAGTAATTTGCTCTATAAATTGGGGCTAGAATG
AATGGTTTGACGAAAATTTAACTGTCTCTATTTTATTTATTAGAAATTAATTTTTATAGTGAAAAAGC
TTAAATTATTTAAAGGGACGAAAAGACCCTATTGAGCTTTATTAATTTATTTTGATTGGGGTGATCAA
GGAATAAAACTTCCTTATTTTTGGAAAAATAAACCAAGTTTTTTGCTTAGAAGATAAGTTACCATAGG
GATAACAGCGTAATTTTTTTTGAGAGTTCATATTGAAAAAGGAGATTGCGACCTCGATGTTGGATTAA
AAAACCTTAAGGTGTAGAGGCTTTATTAGGTAAATCTGTTCGATTTTTAAAATTTT
>defilippi1
AACAGCTGCGGTATTATAACTGTACTAAGGTAGCATAATAATTTGCTCTATAAATTAGGGCTAGAATG
AATGGTTTGACGAGAATTAAACTGTCTCTACTTTATTTGTTAGAAATTAATTTTTATAGTGAAAAAGC
TTGGATGGTTTAAAGGGACGAAAAGACCCTATTGAGCTTTAATAATTTAATTTGGTTGGGGTGATCAA
GGAATAAAACTTCCTTATTAATGGGTTAATAAACCAAGTTTTTTGCTTATAAGGTAAGTTACCATAGG

```
GATAACAGCGTAATTTTTTTGAGAGTTCATATTGAAAAAAAAGATTGCGACCTCGATGTTGGATTAA
AGAACCTTAAGGTGTAGAGGCTTTAATAGGTAAATCTGTTCGATTTTTAAAATTTT
>defilippi2
AACAGCTGCGGTATTATAACTGTACTAAGGTAGCATAATAATTTGCTCTATAAATTAGGGCTAGAATG
AATGGTTTGACGAGAATTAAACTGTCTCTACTTTATTTGTTAGAAATTAATTTTTATAGTGAAAAAGC
TTGGATGGTTTAAAGGGACGAAAAGACCCTATTGAGCTTTAATAATTTAATTTGGTTGGGGTGATCAA
GGAATAAAACTTCCTTATTAATGGGTTAATAAACCAAGTTTTTTGCTTATAAGGTAAGTTACCATAGG
GATAACAGCGTAATTTTTTTGAGAGTTCATATTGAAAAAAAAGATTGCGACCTCGATGTTGGATTAA
AGAACCTTAAGGTGTAGAGGCTTTAATAGGTAAATCTGTTCGATTTTTAAAATTTT
>defilippi3
AACAGCTGCGGTATTATAACTGTACTAAGGTAGCATAATAATTTGCTCTATAAATTAGGGCTAGAATG
AATGGTTTGACGAGAATTAAACTGTCTCTACTTTATTTGTTAGAAATTAATTTTTATAGTGAAAAAGC
TTGGATGGTTTAAAGGGACGAAAAGACCCTATTGAGCTTTAATAATTTAATTTGGTTGGGGTGATCAA
GGAATAAAACTTCCTTATTAATGGGTTAATAAACCAAGTTTTTTGCTTATAAGGTAAGTTACCATAGG
GATAACAGCGTAATTTTTTTGAGAGTTCATATTGAAAAAAAAGATTGCGACCTCGATGTTGGATTAA
AGAACCTTAAGGTGTAGAGGCTTTAATAGGTAAATCTGTTCGATTTTTAAAATTTT
>digueti
AATAGCTGCGGTATTATAACTGTACTAAGGTAGCATAATAATTTGCCTTATAAATTAGGGCTAGAATG
AATGGTTTGACGAGAATTAGACTGTCTCTATTTTATTTATTAGAAATTAATTTTTATAGTGAAAAAGC
TTGGATATATTAAAGGGACGAGAAGACCCTATTGAGCTTTAATGATTTATTTTGATTGGGGTGATCAA
GGAATAAAACTTCCTTATAGATAAATTAATAAACCATATTTTTTGCTTATAAGATAAGTTACCATAGG
GATAACAGCGTAATTTTTTTGAGAGTTCATATTGAAAAAAAAGATTGCGACCTCGATGTTGGATTAA
ATAACCTTAAGGTGAAGAAGCTTTATTAGGTGAATCTGTTCGATTTTTAAAATTTT
>dofleini
AACAGCTGCGGTATTATAACTGTACTAAGGTAGCATAATAATTTGCCTTATAAATTAAGGCTAGAATG
AATGGTTTGACGAAAATTATACTGTCTCTATTTTAATTAATAGAAATTAATTTTTATAGTGAAAAAGC
TTAGATAATTTAAAGGGACGAGAAGACCCTATTGAGCTTTTA?
AATTTATTTTGATTGGGGTGATCAAGGAATAAAACTTCCTTATTTTTAGAATAATTAACCAATGATAT
TGCTTATATGATAAGTTACCATAGGGATAACAGCGTAATTTTTTTGGAGAGTTCATATTAAAAAAAGA
GATTGCGACCTCGATGTTGGATTAAAAAACCATAAGGTGAAGAGGCTTTATTTGGTGAATCTGTTCGA
TTTTTAAAATTTT
>fitchi
AATAGCTGCGGTATTATAACTGTACTAAGGTAGCATAGTAATTTGCCTTATAAATTAGGGCTAGAATG
AATGGTTTGACGAAAATTAGACTGTCTCTATTTTATTTATTAGAAATTAATTTTTATAGTGAGAAAGC
TTGAATAAATTAAAGGGACGAGAAGACCCTATTGAGCTTTATTAATTGATTTTGATTGGGGTGATCAA
GGAATAAAACTTCCTTATTTATAGATTAATAAACCATAATTTTTGCTTAGAAGATAAGTTACCATAGG
GATAACAGCGTAATTTTTTTGAGAGTTCATATTGAAAGAAAAGATTGCGACCTCGATGTTGGATTAA
ATAACCTTAAGGTGAAGAAGCTTTAATAGGTGAATCTGTTCGATTTTTAAAATTTT
>hubbsorum1
AACAGCTGCGGTATTATAACTGTACTAAGGTAGCATAATAATTTGCTCTATAAATTGGGGCTAGAATG
AATGGTTTGACGAAAATTTGACTGTCTCTATCTTATTTATTAGAAATTAATTTTTGTAGTGAGAAAGC
TTAAATTATTTAAAGGGACGAAAAGACCCTATTGAGCTTTAACAATTTATTTTGATTGGGGTGATCAA
GGAATAAAACTTCCTTATTTATAGAAGAATAAACCAAAGTTTTTGCTTAGAAGATAAGTTACCATAGG
GATAACAGCGTAATTTTTTTGAGAGTTCATATTGAAAAAAGAGATTGCGACCTCGATGTTGGATTAA
AAAACCTTAAGGTGAAGAGGCTTTATTAGGTAAATCTGTTCGATTTTTAAAATTTT
>hubbsorum2
AACAGCTGCGGTATTATAACTGTACTAAGGTAGCATAATAATTTGCTCTATAAATTGGGGCTAGAATG
AATGGTTTGACGAAAATTTGACTGTCTCTATCTTATTTATTAGAAATTAATTTTTGTAGTGAGAAAGC
TTAAATTATTTAAAGGGACGAAAAGACCCTATTGAGCTTTAACAATTTATTTTGATTGGGGTGATCAA
GGAATAAAACTTCCTTATTTATAGAAGAATAAACCAAAGTTTTTGCTTAGAAGATAAGTTACCATAGG
GATAACAGCGTAATTTTTTTGAGAGTTCATATTGAAAAAAGAGATTGCGACCTCGATGTTGGATTAA
AAAACCTTAAGGTGAAGAGGCTTTATTAGGTAAATCTGTTCGATTTTTAAAATTTT
>hummelinck1
AACAGCTGCGGTATTATAACTGTACTAAGGTAGCATAATAATTTGCTCTATAAATTGGGGCTAGAATG
```

AATGGTTTGACGAAAATTTGACTGTCTCTATCTTATTTATTAGAAATTAATTTTTGTAGTGAGAAAGC
TTAAATTGTTTAAAGGGACGAAAAGACCCTATTGAGCTTTAATAATTTATTTTGATTGGGGTGATCAA
GGAATAAAACTTCCTTATATATGGAAAAATAAACCAAATTTTTTGCTTAGAAGATAAGTTACCATAGG
GATAACAGCGTAATTTTTTTTGAGAGTTCATATTGAAAAAAGAGATTGCGACCTCGATGTTGGATTAA
AAAACCTTAAGGTGGAGAGGCTTTATTAGGTAAATCTGTTCGATTTTTAAAATTTT
>hummelinck3
AACAGCTGCGGTATTATAACTGTACTAAGGTAGCATAATAATTTGCTCTATAAATTGGGGCTAGAATG
AATGGTTTGACGAAAATTTGACTGTCTCTATCTTATTTATTAGAAATTAATTTTTGTAGTGAGAAAGC
TTAAATTGTTTAAAGGGACGAAAAGACCCTATTGAGCTTTAATAATTTATTTTGATTGGGGTGATCAA
GGAATAAAACTTCCTTATATATGGAAAAATAAACCAAATTTTTTGCTTAGAAGATAAGTTACCATAGG
GATAACAGCGTAATTTTTTTTGAGAGTTCATATTGAAAAAAGAGATTGCGACCTCGATGTTGGATTAA
AAAACCTTAAGGTGGAGAGGCTTTATTAGGTAAATCTGTTCGATTTTTAAAATTTT
>insularis1
AACAGCTGCGGTATTATAACTGTACTAAGGTAGCATAATAATTTGCTCTATAAATTGGGGCTAGAATG
AATGGTTTGACGAAAATTTGACTGTCTCTATTTTATTTATTAGAAATTAATTTTTGTAGTGAGAAAGC
TTAAATTGTTTAAAGGGACGAAAAGACCCTATTGAGCTTTAATAATTTATTTTGATTGGGGTGATCAA
GGAATAAAACTTCCTTATTTGTGGAAAAATAAACCAAATTTTTTGCTTAGAAGATAAGTTACCATAGG
GATAACAGCGTAATTTTTTTTGAGAGTTCATATTGAAAAAAGAGATTGCGACCTCGATGTTGGATTAA
AAAACCTTAAGGTGAAGAGGCTTTATTAGGTAAATCTGTTCGATTTTTAAAATTTT
>insularis4
AACAGCTGCGGTATTATAACTGTACTAAGGTAGCATAATAATTTGCTCTATAAATTGGGGCTAGAATG
AATGGTTTGACGAAAATTTGACTGTCTCTATTTTATTTATTAGAAATTAATTTTTGTAGTGAGAAAGC
TTAAATTGTTTAAAGGGACGAAAAGACCCTATTGAGCTTTAATAATTTATTTTGATTGGGGTGATCAA
GGAATAAAACTTCCTTATTTGTGGAAAAATAAACCAAATTTTTTGCTTAGAAGATAAGTTACCATAGG
GATAACAGCGTAATTTTTTTTGAGAGTTCATATTGAAAAAAGAGATTGCGACCTCGATGTTGGATTAA
AAAACCTTAAGGTGAAGAGGCTTTATTAGGTAAATCTGTTCGATTTTTAAAATTTT
>insularis5
AACAGCTGCGGTATTATAACTGTACTAAGGTAGCATAATAATTTGCTCTATAAATTGGGGCTAGAATG
AATGGTTTGACGAAAATTTGACTGTCTCTATTTTATTTATTAGAAATTAATTTTTGTAGTGAGAAAGC
TTAAATTGTTTAAAGGGACGAAAAGACCCTATTGAGCTTTAATAATTTATTTTGATTGGGGTGATCAA
GGAATAAAACTTCCTTATTTGTGGAAAAATAAACCAAATTTTTTGCTTAGAAGATAAGTTACCATAGG
GATAACAGCGTAATTTTTTTTGAGAGTTCATATTGAAAAAAGAGATTGCGACCTCGATGTTGGATTAA
AAAACCTTAAGGTGAAGAGGCTTTATTAGGTAAATCTGTTCGATTTTTAAAATTTT
>joubini
AATAGCTGCGGTATTATAACTGTACTAAGGTAGCATAATAATTTGCCTTATAAATTAAGGCTAGAATG
AATGGTTTGACGAAAATTAAACTGTCTCTATTTTATTTATTAGAAATTAATTTTTATAGTGAAAAAGC
TTGGATATATTAAAGGGACGAGAAGACCCTATTGAGCTTTGAAAATTTATTTTGATTGGGGTGATCAA
GGAAGAAAACTTCCTTATATATAAATTAATAAACCATAGTTTTTGCTTAGAAGATAAGTTACCATAGG
GATAACAGCGTAATTTTTTTTGAGAGTTCATATTGAGAAAAAAGATTGCGACCTCGATGTTGGATTAA
ATAACCTTAAGGTGGAGAAGCTTTATTGGGTGAATCTGTTCGATTTTTAAAATTTT
>joubini3
AATAGCTGCGGTATTATAACTGTACTAAGGTAGCATAATAATTTGCCTTATAAATTAAGGCTAGAATG
AATGGTTTGACGAAAATTAAACTGTCTCTATTTTATTTATTAGAAATTAATTTTTATAGTGAAAAAGC
TTGGATATATTAAAGGGACGAGAAGACCCTATTGAGCTTTGAAAATTTATTTTGATTGGGGTGATCAA
GGAAGAAAACTTCCTTATATATAAATTAATAAACCATAGTTTTTGCTTAGAAGATAAGTTACCATAGG
GATAACAGCGTAATTTTTTTTGAGAGTTCATATTGAGAAAAAAGATTGCGACCTCGATGTTGGATTAA
ATAACCTTAAGGTGGAGAAGCTTTATTGGGTGAATCTGTTCGATTTTTAAAATTTT
>maya
AACAGCTGCGGTATTATAACTGTACTAAGGTAGCATAATAATTTGCTCTATAAATTGGGGCTAGAATG
AATGGTTTGACGAAAATTTGACTGTCTCTATCTTATTTATTAGAAATTAATTTTTATAGTGAGAAAGC
TTAAATTATTTAAAGGGACGAAAAGACCCTATTGAGCTTTAGTAATTTATTTTGATTGGGGTGATCAA
GGAATAAAACTTCCTTATTTATGGAGAAATAAACCAAATTTTTTGCTTAGATGATAAGTTACCATAGG
GATAACAGCGTAATTTTTTTTGAGAGTTCATATTGAAAAAAGAGATTGCGACCTCGATGTTGGATTAA
AAAACCTTAAGGTGAAGAGGCTTTATTAGGTAAATCTGTTCGATTTTTAAAATTTT

>maya2
AACAGCTGCGGTATTATAACTGTACTAAGGTAGCATAATAATTTGCTCTATAAATTGGGGCTAGAATG
AATGGTTTGACGAAAATTTGACTGTCTCTATCTTATTTATTAGAAATTAATTTTTATAGTGAGAAAGC
TTAAATTATTTAAAGGGACGAAAAGACCCTATTGAGCTTTAGTAATTTATTTTGATTGGGGTGATCAA
GGAATAAAACTTCCTTATTTATGGAGAAATAAACCAAATTTTTTGCTTAGATGATAAGTTACCATAGG
GATAACAGCGTAATTTTTTTTGAGAGTTCATATTGAAAAAAGAGATTGCGACCTCGATGTTGGATTAA
AAAACCTTAAGGTGAAGAGGCTTTATTAGGTAAATCTGTTCGATTTTTAAAATTTT
>mercatoris
AATAGCTGCGGTATTATAACTGTACTAAGGTAGCATAATAATTTGCCTTATAAATTAAGGCTAGAATG
AATGGTTTGACGAGAATTAGACTGTCTCTATTTTATTTAATAGAAATTAATTTTTATGGTGAAAAAGC
TTGAATATATTAAAGGGACGAGAAGACCCTATTGAGCTTTGGGAATTTATTTTGATTGGGGTGATCAA
GGAAGAAAACTTCCTTATATATAAATTAATAAACCATAGTTTTTGCTTAGAAGATAAGTTACCATAGG
GATAACAGCGTAATTTTTTTTGAGAGTTCATATTGAGAAAAAAGATTGCGACCTCGATGTTGGATTAA
ATAACCTTAAGGTGAAGAAGCTTTATTGGGTGAATCTGTTCGATTTTTAAAATTTT
>mimicus
AACAGCTGCGGTATTATAACTGTACTAAGGTAGCATAATAATTTGCTCTATAAATTAGGGCTAGAATG
AATGGTTTGACGAGAATTAAACTGTCTCTATTTTATTTTTTAGAATTTAATTTTTATAGTGAAAAAGC
TTAGATGATTTAAAGGGACGAAAAGACCCTATTGAGCTTTAAGGATTTAATTTGGTTGGGGTGATCAA
GGAATAAAACTTCCTTATTAGTGGGTTAATAAACCAAGTTTTTTGCTTATAAGGTAAGTTACCATAGG
GATAACAGCGTAATTTTTTTTGAGAGTTCATATTGAAAAAAAAGATTGCGACCTCGATGTTGGATTAA
AGAACCTTAAGGTGTAGAGGCTTTAATAGGTAAATCTGTTCGATTTTTAAAATTTT
>mimus1
AACAGCTGCGGTATTATAACTGTACTAAGGTAGCATAATAATTTGCTCTATAAATTGGGGCTAGAATG
AATGGTTTGACGAAAATTTGACTGTCTCTATCTTATTTATTAGAAATTAATTTTTGTAGTGAGAAAGC
TTAAATTATTTAAAGGGACGAAAAGACCCTATTGAGCTTTAACAATTTATTTTGATTGGGGTGATCAA
GGAATAAAACTTCCTTATTTATAGAAGAATAAACCAAAGTTTTTGCTTAGAAGATAAGTTACCATAGG
GATAACAGCGTAATTTTTTTTGAGAGTTCATATTGAAAAAAGAGATTGCGACCTCGATGTTGGATTAA
AAAACCTTAAGGTGAAGAGGCTTTATTAGGTAAATCTGTTCGATTTTTAAAATTTT
>mimus2
AACAGCTGCGGTATTATAACTGTACTAAGGTAGCATAATAATTTGCTCTATAAATTGGGGCTAGAATG
AATGGTTTGACGAAAATTTGACTGTCTCTATCTTATTTATTAGAAATTAATTTTTGTAGTGAGAAAGC
TTAAATTATTTAAAGGGACGAAAAGACCCTATTGAGCTTTAACAATTTATTTTGATTGGGGTGATCAA
GGAATAAAACTTCCTTATTTATAGAAGAATAAACCAAAGTTTTTGCTTAGAAGATAAGTTACCATAGG
GATAACAGCGTAATTTTTTTTGAGAGTTCATATTGAAAAAAGAGATTGCGACCTCGATGTTGGATTAA
AAAACCTTAAGGTGAAGAGGCTTTATTAGGTAAATCTGTTCGATTTTTAAAATTTT
>musakambei
AACAGCTGCGGTATTATAACTGTACTAAGGTAGCATAATAATTTGCCTTATAAATTAGGGCTAGAATG
AATGGTTTGACGAAAATTATACTGTCTCTATTTTAATTAGTAGAAATTAATCTTTATAGTGAAAAAGC
TTAAATAGTTTAAAGGGACGAGAAGACCCTATTGAGCTTTAATAATATATTTTGATTGGGGTGATCAA
GGAATAAAACTTCCTTATTTTTAGAATAATTAACCAATGATATTGCTTATATGATAAGTTACCATAGG
GATAACAGCGTAATTTTTTTGGAGAGTTCATATTAAAAAAAGAGATTGCGACCTCGATGTTGGATTAA
AAAACCATATGGTGAAGAGGCTTTATGTGGTGAATCTGTTCGATTTTTAAAATTTT
>musjanuari
AACAGCTGCGGTATTATAACTGTACTAAGGTAGCATAATAATTTGCCTTATAAATTGAGGCTAGAATG
AATGGTTTGACGAAAATTGTACTGTCTCTATTTTAATTAATAGAAATTAATTTTTATAGTGAAAAAGC
TTAAATAGTTTAAAGGGACGAGAAGACCCTATTGAGCTTTAATAATATATTTTGATTGGGGTGATCAA
GGAATAAAACTTCCTTATTTTTAGAATAATTAACCAATGATATTGCTTATATGATAAGTTACCATAGG
GATAACAGCGTAATTTTTTTGGAGAGTTCATATTAAAAAAAGAGATTGCGACCTCGATGTTGGATTAA
AAAACCATAAGGTGAAGAGGCTTTATATGGTAAATCTGTTCGATTTTTAAAATTTT
>muslonlongi
AACAGCTGCGGTATTATAACTGTACTAAGGTAGCATAATAATTTGCCTTATAAATTAGGGCTAGAATG
AATGGTTTGACGAAAATTATACTGTCTCTATTTTAATTGGTAGAAATTAATCTTTATAGTGAAAAAGC
TTAAATAGTTTAAAGGGACGAGAAGACCCTATTGAGCTTTAATAATATATTTTGATTGGGGTGATCAA
GGAATAAAACTTCCTTATTTTTAGAATAATTAACCAATGATATTGCTTATATGATAAGTTACCATAGG

GATAACAGCGTAATTTTTTTGGAGAGTTCATATTAAAAAAAGAGATTGCGACCTCGATGTTGGATTAA
AAAACCATATGGTGAAGAGGCTTTATGTGGTGAATCTGTTCGATTTTTAAAATTTT
>musyaquinae
AACAGCTGCGGTATTATAACTGTACTAAGGTAGCATAATAATTTGCCTTATAAATTAGGGCTAGAATG
AATGGTTTAACGAAAATTATACTGTCTCTATTTTAATTGATAGAAATTAATTTTTATAGTGAAAAAGC
TTAAATAGTTTAAAGGGACGAGAAGACCCTATTGAGCTTTAATAATATATTTTGATTGGGGTGATCAA
GGAATAAAACTTCCTTATTTTTAGAATAATTAACCAATGATATTGCTTATATGATAAGTTACCATAGG
GATAACAGCGTAATTTTTTTGGAGAGTTCATATTAAAAAAAGAGATTGCGACCTCGATGTTGGATTAA
AAAACCATATGGTGAAGAGGCTTTATATGGTGAATCTGTTCGATTTTTAAAATTTT
>oculifer
AACAGCTGCGGTATTATAACTGTACTAAGGTAGCATAATAATTTGCTCTATAAATTGGGGCTAGAATG
AATGGTTTGACGAAAATTTGGCTGTCTCTGTTTTATTTATTAGAAATTAATTTTTGTAGTGAAAAAGC
TTAAATTATTTAAAGGGACGAAAAGACCCTATTGAGCTTTAATAATTTATTTTGATTGGGGTGATCGA
GGAATAAAACTTCCTTATTTTTGGAAAAATAAACCAAATTTTTTGCTTAGAAGATAAGTTACCATAGG
GATAACAGCGTAATTTTTTTTGAGAGTTCATATTGAAAAAAGAGATTGCGACCTCGATGTTGGATTAA
AAAACCTTAAGGTGGAGAGGCTTTATTAGGTAAATCTGTTCGATTTTTAAAATTTT
>ornatus
AACAGCTGCGGTATTATAACTGTACTAAGGTAGCATAATAATTTGCCTTGTAAATTAAGGCTAGAATG
AATGGTTTGACGAAAATTAAGCTGTCTCTGATTTATTTGTTAGAAATTAATTTTTATAGTGAAAAAGC
TTGGATAATTTAAAGGGACGAGAAGACCCTATTGAGCTTTAATAATATATTTTGATTGGGGTGATCAA
GGAATAAAACTTCCTTATTAATTGAGTAATAAACCAAAGTTTTTGCTTATAAGATAAGTTACCATAGG
GGTAACAGCGTAATTTTTTTTGAGAGTTCATATTGAAAAAAGAGATTGCGACCTCGATGTTGGATTAA
AATACCTTAAGGTGAAGAGGCTTTATTAGGTGAATCTGTTCGATTTTTAAAATTTT
>tetricus1
AACAGCTGCGGTATTATAACTGTACTAAGGTAGCATAATAATTTGCCTTATAAATTGGGGCTAGAATG
AATGGTTTGACGAGAATTTGACTGTCTCTATTTTATTTATTAGAAATTAATTTTTATAGTGAAAAAGC
TTTAATTATTTAAAGGGACGAAAAGACCCTATTGAGCTTTAATAATTTACTTTGGTTGGGGTGATCAA
GGAATAAAACTTCCTTATTAATTGAAGAATAAACCAAGTTTTTTGCTTAGAAGATAAGTTACCATAGG
GATAACAGCGTAATTTTTTTTGAGAGTTCATATTGAAAAAAGAGATTGCGACCTCGATGTTGGATTAA
AAGACCTTAAGGTGTAGAGGCTTTATTAGGTAAATCTGTTCGATTTTTAAAATTTT
>tetricus2
AACAGCTGCGGTATTATAACTGTACTAAGGTAGCATAATAATTTGCCTTATAAATTGGGGCTAGAATG
AATGGTTTGACGAGAATTTGACTGTCTCTATTTTATTTATTAGAAATTAATTTTTATAGTGAAAAAGC
TTTAATTATTTAAAGGGACGAAAAGACCCTATTGAGCTTTAATAATTTACTTTGGTTGGGGTGATCAA
GGAATAAAACTTCCTTATTAATTGAAGAATAAACCAAGTTTTTTGCTTGGAAGATAAGTTACCATAGG
GATAACAGCGTAATTTTTTTTGAGAGTTCATATTGAAAAAAGAGATTGCGACCTCGATGTTGGATTAA
AAGACCTTAAGGTGTAGAGGCTTTATTAGGTAAATCTGTTCGATTTTTAAAATTTT
>vulgaris1
AACAGCTGCGGTATTATAACTGTACTAAGGTAGCATAATAATTTGCCCTATAAATTGGGGCTAGAATG
AATGGTTTGACGAGAATTTAACTGTCTCTATTTTATTTATTAGAAATTAATTTTTATAGTGAAAAAGC
TTTAATTGTTTAAAGGGACGAAAAGACCCTATTGAGCTTTAATAGTTTACTTTGGTTGGGGTGATCAA
GGAATAAAACTTCCTTATTAGTTGAAGAATAAACCAAGTTTTTTGCTTAGAAGATAAGTTACCATAGG
GATAACAGCGTAATTTTTTTTGAGAGTTCATATTGAAAAAAGAGATTGCGACCTCGATGTTGGATTAA
AAGACCTTAAGGTGTAGAGGCTTTATTAGGTAAATCTGTTCGATTTTTAAAATTTT
>vulgaris2
AACAGCTGCGGTATTATAACTGTACTAAGGTAGCATAATAATTTGCCCTATAAATTGGGGCTAGAATG
AATGGTTTGACGAGAATTTGACTGTCTCTATTTTATTTATTAGAAATTAATTTTTATAGTGAAAAAGC
TTTGATTATTTAAAGGGACGAAAAGACCCTATTGAGCTTTGATAGTTTACTTTGGTTGGGGTGATCAA
GGAATAAAACTTCCTTATTAGTTGAAGAATAAACCAAGTTTTTTGCTTAGAAGATAAGTTACCATAGG
GATAACAGCGTAATTTTTTTTGAGAGTTCATATTGAAAAAAGAGATTGCGACCTCGATGTTGGATTAA
AAGACCTTAAGGTGTAGAGGCTTTATTAGGTAAATCTGTTCGATTTTTAAAATTTT
>vulgaris3
AACAGCTGCGGTATTATAACTGTACTAAGGTAGCATAATAATTTGCCCTATAAATTGGGGCTAGAATG
AATGGTTTGACGAGAATTTGACTGTCTCTATTTTATTTATTAGAAATTAATTTTTATAGTGAAAAAGC

```
TTTGATTATTTAAAGGGACGAAAAGACCCTATTGAGCTTTGATAGTTTACTTTGGTTGGGGTGATCAA
GGAATAAAACTTCCTTATTAGTTGAAGAATAAACCAAGTTTTTTGCTTAGAAGATAAGTTACCATAGG
GATAACAGCGTAATTTTTTTTGAGAGTTCATATTGAAAAAAGAGATTGCGACCTCGATGTTGGATTAA
AAGACCTTAAGGTGTAGAGGCTTTATTAGGTAAATCTGTTCGATTTTTAAAATTTT
>vulgaris4
AACAGCTGCGGTATTATAACTGTACTAAGGTAGCATAATAATTTGCCCTATAAATTGGGGCTAGAATG
AATGGTTTGACGAGAATTTGACTGTCTCTATTTTATTTATTAGAAATTAATTTTTATAGTGAAAAAGC
TTTGATTATTTAAAGGGACGAAAAGACCCTATTGAGCTTTGATAGTTTACTTTGGTTGGGGTGATCAA
GGAATAAAACTTCCTTATTAGTTGAAGAATAAACCAAGTTTTTTGCTTAGAAGATAAGTTACCATAGG
GATAACAGCGTAATTTTTTTTGAGAGTTCATATTGAAAAAAGAGATTGCGACCTCGATGTTGGATTAA
AAGACCTTAAGGTGTAGAGGCTTTATTAGGTAAATCTGTTCGATTTTTAAAATTTT
>whitev
AACAGCTGCGGTATTATAACTGTACTAAGGTAGCATAATAATTTGCTCTATAAATTAGGGCTAGAATG
AATGGTTTGACGAGAATTAAACTGTCTCTATTTTATTTATTAGAAATTAATTTTTATAGTGAGAAAGC
TTAAATGGTTTAAAGGGACGAAAAGACCCTATTGAGCTTTAATAATATAATTTGATTGGGGTGATCAA
GGAATAAAACTTCCTTATTAATGGGTTAATAAACCAAGTTTTTTGCTTAAAAGATAAGTTACCATAGG
GATAACAGCGTAATTTTTTTTGAGAGTTCATATTGAAAAAAGAGATTGCGACCTCGATGTTGGATTAA
AGAACCTTAAGGTGTAGAGGCTTTAATAGGTAAATCTGTTCGATTTTTAAAATTTT
```
