# Supplementary material for: A biogeographic framework of octopod species diversification: the role of the Isthmus of Panama
Source: PeerJ. 2020 Mar 27;8:e8691. doi: 10.7717/peerj.8691 (PMC7104719; doi:10.7717/peerj.8691)
Supplement: Supplemental Information 4 [file peerj-08-8691-s004.pdf]

>383
CGTACCGAATTAGGTCAACCAGGCTCACTCCTCAATGACGACCAACTTTACAATGTAATTGTAACTGC
ACATGCATTTGTCATAATTTTTTTTTTAGTTATACCTGTTATAATTGGAGGATTTGGAAATTGATTAG
TACCTTTAATATTAGGAGCCCCAGATATAGCATTTCCCCGAATAAATAATATAAGTTTCTGACTTCTC
CCTCCTTCCTTAACCTTATTATTGACTTCAGCAGCTGTCGAAAGAGGAGTGGGAACAGGTTGAACTGT
TTACCCCCCTCTATCAAGAAATCTCGCTCACACAGGCCCATCTGTAGATTTAGCAATTTTTTCACTTC
ATTTAGCTGGAATTTCATCCATTTTAGGAGCTATTAATTTCATTACTACAATCATTAATATACGATGA
GAAGGAATACAAATAGAACGTCTTCCTTTATTTGTTTGATCTGTATTTATTACAGCAATTCTTTTACT
TTTATCTTTGCCTGTTCTCGCCGGAGCCATTACTATATTATTAACTGACCGAAATTTCAATACTACAT
TTTTTGATCCTAGAGGAGGA
>384
CGTACTGAATTAGGTCAACCAGGCTCACTCCTCAATGACGATCAACTTTACAACGTAATTGTAACTGC
ACATGCATTTGTCATAATTTTTTTTTTAGTTATACCTGTAATAATTGGAGGATTTGGAAATTGATTAG
TACCTTTAATACTAGGAGCCCCAGATATAGCATTCCCCCGAATAAATAATATAAGTTTCTGACTTCTT
CCTCCTTCCTTAACCTTATTACTGACCTCAGCAGCCGTTGAAAGAGGAGTGGGAACAGGTTGAACTGT
ATACCCTCCTCTATCAAGAAATCTCGCTCACACAGGCCCATCTGTAGATTTAGCAATTTTTTCACTTC
ATTTAGCTGGAATTTCATCCATTTTAGGAGCTATTAATTTCATTACTACAATTATTAATATACGATGG
GAAGGAATACAAATAGAACGTCTTCCTTTATTTGTTTGATCTGTATTTATTACAGCAATTCTTTTACT
TTTATCTTTACCCGTTCTCGCTGGAGCCATTACTATATTATTAACTGACCGAAATTTCAATACTACAT
TTTTTGATCCTAGAGGAGGA
>385
CGTACTGAATTAGGTCAACCAGGCTCACTCCTCAATGACGATCAACTTTACAACGTAATTGTAACTGC
ACATGCATTTGTCATAATTTTTTTTTTAGTTATACCTGTAATAATTGGAGGATTTGGAAATTGATTAG
TACCTTTAATACTAGGAGCCCCAGATATAGCATTCCCCCGAATAAATAATATAAGTTTCTGACTTCTT
CCTCCTTCCTTAACCTTATTACTGACCTCAGCAGCCGTTGAAAGAGGAGTGGGAACAGGTTGAACTGT
ATACCCTCCTCTATCAAGAAATCTCGCTCACACAGGCCCATCTGTAGATTTAGCAATTTTTTCACTTC
ATTTAGCTGGAATTTCATCCATTTTAGGAGCTATTAATTTCATTACTACAATTATTAATATACGATGG
GAAGGAATACAAATAGAACGTCTTCCTTTATTTGTTTGATCTGTATTTATTACAGCAATTCTTTTACT
TTTATCTTTACCCGTTCTCGCTGGAGCCATTACTATATTATTAACTGACCGAAATTTCAATACTACAT
TTTTTGATCCTAGAGGAGGA
>386
CGTACTGAATTAGGTCAACCAGGCTCACTCCTCAATGACGATCAACTTTACAACGTAATTGTAACTGC
ACATGCATTTGTCATAATTTTTTTTTTAGTTATACCTGTAATAATTGGAGGATTTGGAAATTGATTAG
TACCTTTAATACTAGGAGCCCCAGATATAGCATTCCCCCGAATAAATAATATAAGTTTCTGACTTCTT
CCTCCTTCCTTAACCTTATTACTGACCTCAGCAGCCGTTGAAAGAGGAGTGGGAACAGGTTGAACTGT
ATACCCTCCTCTATCAAGAAATCTCGCTCACACAGGCCCATCTGTAGATTTAGCAATTTTTTCACTTC
ATTTAGCTGGAATTTCATCCATTTTAGGAGCTATTAATTTCATTACTACAATTATCAATATACGATGG
GAAGGAATACAAATAGAACGTCTTCCTTTATTTGTTTGATCTGTATTTATTACAGCAATTCTTTTACT
TTTATCTTTACCCGTTCTCGCTGGAGCCATTACTATATTATTAACTGACCGAAATTTCAATACTACAT
TTTTTGATCCTAGAGGAGGA
>bimaculatus
CGAACAGAATTAGGTCAACCAGGATCTCTTCTCAATGATGATCAATTATATAATGTTATTGTTACAGC
CCATGCATTTGTAATAATCTTTTTTCTTGTTATACCTGTCATAATTGGAGGATTTGGCAACTGATTAG
TTCCCTTAATATTGGGAGCCCCAGATATAGCATTCCCACGAATAAATAACATAAGTTTTTGACTTTTA
CCACCCTCTCTCACTTTATTATTATCCTCAGCCGCAGTAGAAAGAGGTGTAGGAACTGGATGAACCGT
ATATCCCCCTCTCTCAAGAAACTTAGCTCATATAGGTCCATCCGTTGATTTAGCTATTTTTTCACTCC
ACTTAGCCGGTATCTCATCTATCCTTGGAGCTATTAATTTTATTACAACTATTATTAATATACGATGA
GAAGGAATACTAATAGAACGACTCCCATTATTTGTATGATCAGTATTTATTACTGCAATTTTATTATT
ACTTTCTCTACCAGTTCTTGCTGGAGCAATTACAATACTTTTAACTGATCGAAATTTTAATACCACTT
TTTTTGATCCGAGAGGGGGG
>bimaculoides1
CGAACAGAATTAGGTCAACCAGGATCTCTTCTCAATGATGATCAATTATATAATGTTATTGTCACAGC
CCATGCATTTGTAATAATCTTTTTCCTTGTTATGCCCGTTATAATTGGAGGATTCGGCAACTGATTAG
TTCCCTTAATATTAGGAGCCCCAGACATAGCATTTCCACGAATAAATAATATAAGTTTTTGACTTCTA

CCACCCTCTCTTACTTTATTACTATCCTCAGCTGCAGTAGAAAGAGGTGTAGGAACCGGATGAACCGT
ATACCCTCCTCTTTCAAGAAATTTAGCTCATATAGGTCCATCCGTTGATTTAGCTATTTTTTCACTAC
ATCTAGCTGGTATCTCATCTATTCTTGGAGCTATTAATTTTATTACAACTATCATTAATATACGATGA
GAAGGAATACTAATAGAACGACTCCCATTATTTGTATGATCAGTATTTATTACTGCAATTTTATTATT
ACTTTCCTTACCAGTCCTTGCCGGAGCAATTACAATACTTTTAACTGATCGAAATTTTAATACCACTT
TCTTTGACCCAAGAGGAGGA
>bimaculoides2
CGAACAGAATTAGGTCAACCAGGATCTCTTCTCAATGATGATCAATTATATAATGTTATTGTCACAGC
CCATGCATTTGTAATAATCTTTTTCCTTGTTATGCCCGTTATAATTGGAGGATTCGGCAACTGATTAG
TTCCCTTAATATTAGGAGCCCCAGACATAGCATTTCCACGAATAAATAATATAAGTTTTTGACTTCTA
CCACCCTCTCTTACTTTATTACTATCCTCAGCTGCAGTAGAAAGAGGTGTAGGAACCGGATGAACCGT
ATACCCTCCTCTTTCAAGAAATTTAGCTCATATAGGTCCATCCGTTGATTTAGCTATTTTTTCACTAC
ATCTAGCTGGTATCTCATCTATTCTTGGAGCTATTAATTTTATTACAACTATCATTAATATACGATGA
GAAGGAATACTAATAGAACGACTCCCATTATTTGTATGATCAGTATTTATTACTGCAATTTTATTATT
ACTTTCCTTACCAGTCCTTGCCGGAGCAATTACAATACTTTTAACTGATCGAAATTTTAATACCACTT
TCTTTGACCCAAGAGGAGGA
>briareus
CGAACAGAATTAGGACAACCTGGTTCCCTCCTTAATGATGATCAACTATATAATGTAATCGTCACAGC
TCATGCATTCGTAATAATTTTCTTCCTTGTTATACCAGTCATAATTGGAGGATTTGGAAATTGATTAG
TACCATTAATATTAGGTGCCCCTGATATAGCATTCCCTCGAATAAATAATATAAGCTTTTGACTTCTC
CCCCCATCATTAACTCTCTTATTATCTTCTGCTGCAGTAGAAAGTGGTGTTGGAACTGGATGAACCGT
TTACCCCCCTCTTTCAAGAAATTTAGCTCATATAGGACCATCTGTCGATTTAGCTATTTTTTCACTTC
ATCTAGCAGGTATTTCTTCAATTCTTGGAGCTATTAACTTTATCACAACTATTATTAATATACGATGA
GAAGGAATATTTATAGAACGACTTCCACTATTTGTATGATCCGTCTTCATTACTGCTATTCTACTTCT
CCTTTCATTACCAGTTCTTGCTGGAGCAATTACTATACTCCTAACCGATCGAAATTTTAATACCACAT
TTTTTGATCCTAGAGGAGGA
>briareus2
CGAACAGAATTAGGACAACCTGGTTCCCTCCTTAATGATGATCAACTATATAATGTAATCGTCACAGC
TCATGCATTCGTAATAATTTTCTTCCTTGTTATACCAGTCATAATTGGAGGATTTGGAAACTGATTAG
TACCATTAATATTAGGTGCCCCTGATATAGCATTCCCTCGAATAAATAATATAAGCTTTTGACTTCTC
CCCCCATCATTAACTCTCTTATTATCTTCTGCTGCAGTAGAAAGTGGTGTTGGAACTGGATGAACCGT
TTACCCCCCTCTTTCAAGAAATTTAGCTCATATAGGACCATCTGTCGATTTAGCTATTTTTTCACTTC
ATCTAGCAGGTATTTCTTCAATTCTTGGAGCTATTAACTTTATCACAACTATTATTAATATACGATGA
GAAGGAATACTTATAGAACGACTTCCACTATTTGTATGATCCGTCTTCATTACTGCTATTCTACTTCT
GCTTTCATTACCAGTTCTTGCTGGAGCAATTACTATACTCCTAACCGATCGAAATTTTAATACCACAT
TTTTTGATCCTAGAGGAGGA
>defilippi1
CGAACAGAATTAGGACAACCAGGATCTCTCTTAAATGATGACCAATTATATAATGTAATTGTAACAGC
TCATGCATTCGTAATAATTTTTTTTCTAGTTATACCTGTAATAATTGGAGGATTTGGAAATTGATTAG
TGCCTTTGATATTAGGAGCTCCTGATATAGCTTTCCCTCGAATAAATAACATAAGATTTTGATTACTT
CCCCCCTCATTAACACTACTTTTATCTTCAGCTGCAGTTGAAAGAGGTGTAGGAACCGGATGAACTGT
ATACCCTCCACTCTCAAGAAATCTAGCTCACATAGGACCCTCAGTCGATCTTGCTATTTTTTCTCTTC
ATTTAGCTGGTATTTCGTCAATTCTTGGAGCCATTAATTTTATTACTACTATTATTAATATACGATGA
GAAGGAATATTAATAGAACGTCTCCCCTTATTCGTCTGATCTGTTTTTATTACAGCAATTCTACTACT
CTTATCACTCCCAGTTTTAGCAGGTGCGATTACTATATTATTAACAGATCGAAACTTTAACACTACAT
TTTTTGATCCAAGAGGTGGA
>defilippi2
CGAACAGAATTAGGACAACCAGGATCTCTCTTAAATGATGACCAATTATATAATGTAATTGTAACAGC
TCATGCATTCGTAATAATTTTTTTTCTAGTTATACCTGTAATAATTGGAGGATTTGGAAATTGATTAG
TGCCTTTGATATTAGGAGCTCCTGATATAGCTTTCCCTCGAATAAATAACATAAGATTTTGATTACTT
CCCCCCTCATTAACACTACTTTTATCTTCAGCTGCAGTTGAAAGAGGTGTAGGAACCGGATGAACTGT
ATACCCTCCACTCTCAAGAAATCTAGCTCACATGGGACCCTCAGTCGATCTTGCTATTTTTTCTCTTC
ATTTAGCTGGTATTTCGTCAATTCTTGGAGCCATTAATTTTATTACTACTATTATTAATATACGATGA
GAAGGAATATTAATAGAACGTCTCCCCTTATTCGTCTGATCTGTTTTTATTACAGCAATTCTACTACT

CTTATCACTCCCAGTTTTAGCAGGTGCGATTACTATATTATTAACAGATCGAAACTTTAACACTACAT
TTTTTGATCCAAGAGGTGGA
>defilippi3
CGAACAGAATTAGGACAACCAGGATCTCTCTTAAATGATGACCAATTATATAATGTAATTGTAACAGC
TCATGCATTCGTAATAATTTTTTTTCTAGTTATACCTGTAATAATTGGAGGGTTTGGAAATTGATTAG
TGCCTTTGATATTAGGAGCTCCTGATATAGCTTTCCCTCGAATAAATAACATAAGATTTTGATTACTT
CCCCCCTCATTAACACTACTTTTATCTTCAGCTGCAGTTGAAAGAGGTGTAGGAACCGGATGAACTGT
ATATCCTCCACTCTCAAGAAATCTAGCTCACATGGGACCCTCAGTCGATCTTGCTATTTTTTCTCTTC
ATTTAGCTGGTATTTCGTCAATTCTTGGAGCCATTAATTTTATTACTACTATTATTAATATACGATGA
GAAGGAATATTAATAGAACGTCTCCCCTTATTCGTCTGATCTGTTTTTATTACAGCAATTCTACTACT
CCTATCACTCCCAGTTTTAGCAGGTGCGATTACTATATTATTAACAGACCGAAACTTTAACACTACAT
TTTTTGATCCAAGAGGTGGA
>digueti
CGAACAGAATTAGGTCAACCAGGATCCCTATTAAATGATGATCAATTATACAATGTAATCGTAACTGC
TCATGCTTTTGTCATAATTTTTTTTTTAGTTATACCAGTAATAATTGGAGGATTTGGTAACTGATTAG
TTCCACTAATATTAGGAGCTCCAGATATAGCATTCCCTCGTATAAATAATATAAGATTTTGATTACTT
CCCCCTTCATTAACATTACTCCTTACCTCAGCTGCTGTTGAAAGTGGTGTAGGAACTGGATGAACTGT
TTACCCCCCTCTCTCTAGAAATCTAGCTCATATAGGTCCCTCAGTCGATCTCGCTATTTTCTCGCTCC
ATTTAGCAGGAATTTCATCTATTCTAGGAGCTATTAACTTTATTACTACTATTATTAATATACGATGA
GAAGGAATATTAATGGAACGTCTTCCTTTATTTGTTTGATCAGTTTTTATTACTGCAATTTTATTACT
TTTATCTCTTCCAGTTCTTGCTGGAGCTATTACTATATTACTTACTGATCGTAATTTTAACACCACTT
TTTTTGATCCAAGAGGAGGA
>dofleini
CGAACAGAACTAGGACAACCTGGATCTTTACTAAATGATGATCAACTTTATAACGTCATTGTTACTGC
CCACGCTTTTGTAATAATTTTCTTTTTAGTTATACCCGTAATAATTGGAGGATTTGGAAACTGATTAG
TCCCTTTAATATTAGGAGCTCCAGATATAGCATTCCCACGAATAAACAATATAAGATTTTGATTACTT
CCCCCCTCTTTAACTCTCCTATTAACTTCAGCAGCAGTAGAAAGAGGAGCAGGAACAGGTTGAACTGT
ATACCCTCCATTATCTAGAAATTTAGCCCATATAGGTCCTTCTGTAGATCTAGCAATTTTTTCTCTTC
ATTTAGCAGGTATTTCCTCTATTTTAGGAGCTATTAATTTCATCACAACTATTATTAATATACGATGA
GAAGGGATACAAATAGAACGTCTTCCACTATTTGTATGATCTGTTCTAATTACAGCAGTTCTTCTTCT
ATTATCTTTACCAGTATTAGCAGGTGCCATTACTATACTTCTTACTGATCGTAACTTCAATACAACTT
TTTTTGACCCAAGAGGAGGG
>fitchi
CGAACAGAATTAGGCCAACCAGGATCTTTATTAAATGACGATCAATTATATAATGTAATTGTAACTGC
TCATGCATTCGTTATAATTTTCTTTTTAGTTATACCAGTAATGATTGGTGGATTTGGTAATTGATTAG
TACCATTAATATTAGGAGCTCCAGATATAGCTTTCCCTCGAATAAATAATATAAGCTTTTGATTACTT
CCCCCTTCATTAACCTTACTTCTTTGTTCAGCTGCGGTTGAAAGAGGGGTAGGTACAGGATGAACTGT
CTACCCCCCTTTATCAAGAAATTTAGCTCATATAGGTCCTTCTGTTGATCTTGCTATTTTCTCTTTAC
ATTTAGCAGGAATTTCATCTATTTTAGGAGCAATTAACTTCATCACTACTATTATTAATATACGATGA
GAAGGTATACTTATAGAACGACTTCCTTTATTTGTTTGATCCGTATTTATTACCGCAATTTTATTACT
TCTATCCCTTCCTGTTCTTGCTGGAGCCATTACTATACTTTTAACCGACCGTAATTTTAACACTACCT
TTTTTGACCCTAGAGGAGGA
>hubbsorum1
CGAACAGAATTAGGTCAACCAGGATCTCTCCTAAATGATGATCAACTATATAACGTAATTGTTACAGC
ACATGCATTTGTAATAATTTTTTTTCTTGTTATACCTGTTATAATTGGAGGATTTGGTAATTGATTAG
TTCCCTTAATACTAGGAGCTCCAGACATAGCATTCCCACGAATAAACAATATAAGTTTTTGACTTTTA
CCACCTTCTCTTACCCTATTATTATCTTCAGCTGCAGTAGAAAGAGGTGCAGGTACTGGATGGACTGT
ATATCCCCCTCTTTCAAGAAATTTAGCTCATATAGGCCCTTCCGTTGATCTAGCTATTTTTTCTCTTC
ATTTAGCTGGTATTTCATCTATTCTTGGAGCCATTAACTTCATTACAACTATTATCAATATACGATGA
GAAGGAATATTAATAGAACGACTTCCATTATTTGTATGATCAGTATTTATTACCGCAATTTTATTACT
TCTTTCCTTACCAGTCCTTGCTGGAGCAATTACAATACTCTTAACAGATCGAAATTTTAATACCACTT
TTTTTGACCCAAGGGGAGGA
>hubbsorum2
CGAACAGAATTAGGTCAACCAGGATCTCTCCTAAATGATGATCAACTATATAACGTAATTGTTACAGC

ACATGCATTTGTAATAATTTTTTTTCTTGTTATACCTGTTATAATTGGAGGATTTGGTAATTGATTAG
TTCCCTTAATACTAGGAGCTCCAGACATAGCATTCCCACGAATAAACAATATAAGTTTTTGACTTTTA
CCACCTTCTCTTACCCTATTATTATCTTCAGCTGCAGTAGAAAGAGGTGCAGGTACTGGATGGACTGT
ATATCCCCCTCTTTCAAGAAATTTAGCTCATATAGGCCCTTCCGTTGATCTAGCTATTTTTTCTCTTC
ATTTAGCTGGTATTTCATCTATTCTTGGAGCCATTAACTTCATTACAACTATTATCAATATACGATGA
GAAGGAATATTAATAGAACGACTTCCATTATTTGTATGATCAGTATTTATTACCGCAATTTTATTACT
TCTTTCCTTACCAGTCCTTGCTGGAGCAATTACAATACTCTTAACAGATCGAAATTTTAATACCACTT
TTTTTGACCCAAGGGGAGGA
>hummelinck1
CGAACAGAATTAGGACAACCAGGATCTCTTCTCAATGATGATCAATTATATAATGTAATTGTTACAGC
ACATGCATTTGTAATAATTTTTTTTCTAGTTATACCTGTTATAATTGGAGGATTTGGCAATTGATTAG
TCCCCTTAATATTAGGAGCTCCAGATATAGCATTTCCACGAATAAATAATATAAGTTTTTGACTTTTA
CCACCTTCTCTTACCCTATTACTATCTTCAGCTGCAGTAGAAAGAGGGGCAGGTACTGGATGAACTGT
ATATCCTCCTCTTTCAAGAAACTTAGCTCATATAGGACCTTCCGTCGATCTAGCTATTTTTTCTCTTC
ACTTAGCTGGTATTTCATCCATTCTTGGAGCCATTAATTTTATCACAACTATTATCAATATACGATGA
GAAGGAATATTAATAGAACGACTCCCATTATTTGTGTGAGCAGTATTTATTACCGCAATTCTACTACT
ACTTTCCTTACCAGTTCTTGCTGGAGCAATTACAATACTTCTAACAGACCGAAATTTTAATACCACTT
TTTTCGATCCAAGAGGAGGT
>hummelinck3
CGAACAGAACTAGGACAACCAGGATCTCTTCTCAATGATGATCAATTATATAATGTAATTGTTACAGC
ACATGCATTTGTAATAATTTTTTTTCTAGTTATACCTGTTATAATTGGAGGATTTGGTAATTGATTAG
TCCCCTTAATATTAGGAGCTCCAGATATAGCATTTCCACGAATAAATAATATAAGTTTTTGACTTTTA
CCACCTTCTCTTACCCTATTATTATCTTCAGCTGCAGTAGAAAGAGGGGCAGGTACTGGATGAACTGT
ATATCCTCCTCTTTCAAGAAACTTAGCTCATATAGGACCTTCCGTCGATCTAGCTATTTTTTCTCTTC
ACTTAGCTGGTATTTCATCCATTCTTGGAGCCATTAATTTTATCACAACTATTATCAATATACGATGA
GAAGGAATATTAATAGAACGACTCCCATTATTTGTGTGAGCAGTATTTATTACCGCAATTCTACTACT
ACTTTCCTTACCAGTTCTTGCTGGAGCAATTACAATACTTCTAACAGACCGAAATTTTAATACCACTT
TTTTCGATCCAAGAGGAGGT
>insularis1
CGAACAGAATTAGGTCAACCAGGATCTCTTCTTAATGATGACCAACTATACAATGTAATTGTAACAGC
ACATGCATTTGTAATAATTTTTTTTCTAGTTATACCTGTTATAATTGGAGGATTTGGTAATTGATTAG
TTCCTTTAATACTAGGAGCCCCAGATATAGCATTCCCACGAATAAATAACATAAGTTTTTGACTTTTA
CCACCTTCTCTTACCCTATTATTATCTTCAGCCGCAGTAGAAAGAGGTGCAGGTACCGGATGAACTGT
ATACCCTCCTCTTTCAAGAAATTTAGCCCATATAGGTCCTTCCGTTGATCTAGCTATTTTCTCCCTTC
ATTTAGCTGGTATTTCATCCATTCTTGGAGCCATTAATTTTATTACAACTATTATCAATATACGATGA
GAAGGAATATTAATAGAACGACTCCCATTATTTGTATGAGCAGTATTTATTACTGCAATTTTATTACT
ACTCTCCTTACCAGTCCTCGCTGGAGCAATTACTATACTTTTAACAGATCGAAATTTTAATACCACTT
TTTTTGACCCAAGAGGTGGT
>insularis4
CGAACAGAATTAGGTCAACCAGGATCTCTTCTTAATGATGACCAACTATACAATGTAATTGTAACAGC
ACATGCATTTGTAATAATTTTTTTTCTAGTTATACCTGTTATAATTGGAGGATTTGGTAATTGATTAG
TTCCTTTAATACTAGGAGCCCCAGATATAGCATTCCCACGAATAAATAACATAAGTTTTTGACTTTTA
CCACCTTCTCTTACCCTATTATTATCTTCAGCCGCAGTAGAAAGAGGTGCAGGTACCGGATGAACTGT
ATACCCTCCCCTTTCAAGAAATTTAGCCCATATAGGTCCTTCCGTTGATCTAGCTATTTTCTCCCTTC
ATTTAGCTGGTATTTCATCCATTCTTGGAGCCATTAATTTTATTACAACTATTATCAATATACGATGA
GAAGGAATATTAATAGAACGACTCCCATTATTTGTATGAGCAGTATTTATTACTGCAATTTTATTACT
ACTCTCCTTACCAGTCCTCGCTGGAGCAATTACTATACTTTTAACAGATCGAAATTTTAATACCACTT
TTTTTGACCCAAGAGGTGGT
>insularis5
CGAACAGAATTAGGTCAACCAGGATCTCTTCTTAATGATGACCAACTATACAATGTAATTGTAACAGC
ACATGCATTTGTAATAATTTTTTTTCTAGTTATACCTGTTATAATTGGAGGATTTGGTAATTGATTAG
TTCCTTTAATACTAGGAGCCCCAGATATAGCATTCCCACGGATAAATAACATAAGTTTTTGACTTTTA
CCTCCTTCTCTTACCCTATTATTATCTTCAGCCGCAGTAGAAAGAGGTGCAGGTACCGGATGAACTGT
ATATCCTCCTCTTTCAAGAAATTTAGCCCATATAGGTCCTTCCGTTGATCTAGCTATTTTCTCCCTTC

ATTTAGCTGGTATTTCATCCATTCTTGGAGCCATTAATTTTATTACAACTATTATCAATATACGATGA
GAAGGAATATTAATAGAACGACTCCCATTATTTGTATGAGCAGTATTTATTACTGCAATTTTATTACT
ACTCTCCTTACCAGTCCTCGCTGGAGCAATTACTATACTTTTAACAGATCGAAATTTTAATACCACTT
TTTTTGACCCAAGAGGTGGT
>joubini
CGAACAGAATTAGGCCAACCAGGATCTTTATTAAATGATGATCAATTATATAATGTAATTGTAACCGC
TCATGCTTTTGTTATAATTTTTTTTTTGGTTATACCAATTATAATTGGAGGATTTGGAAACTGACTAG
TCCCCTTAATATTAGGAGCACCAGATATAGCTTTTCCTCGAATAAACAATATAAGATTTTGATTATTA
CCTCCCTCTTTAACATTACTACTTACTTCAGCTGCTGTTGAAAGAGGTGTAGGAACTGGATGAACTGT
ATACCCTCCTCTTTCTAGAAACTTAGCTCATATAGGCCCTTCAGTAGATCTTGCTATTTTTTCTCTTC
ACTTAGCTGGAATCTCTTCTATTTTAGGAGCTATTAATTTTATCACCACCATTATTAATATACGCTGA
GAAGGAATATTAATAGAACGACTTCCTTTATTTGTTTGATCAGTTTTTATTACAGCTGTATTATTACT
ATTATCCCTTCCTGTTCTAGCTGGAGCTATTACTATACTTCTTACCGATCGTAATTTTAATACTACTT
TTTTTGATCCAAGAGGAGGA
>joubini3
CGAACAGAATTAGGTCAACCAGGATCTTTATTAAATGATGATCAACTATATAATGTAATTGTAACCGC
TCATGCTTTTGTTATAATTTTTTTTTTGGTTATACCAGTTATAATTGGAGGATTTGGAAATTGATTAG
TACCTTTAATACTAGGAGCTCCAGATATAGCTTTCCCCCGTATAAATAATATAAGATTTTGATTACTT
CCACCCTCATTAACACTATTACTTACTTCAGCTGCTGTTGAAAGAGGTGTAGGAACTGGATGAACTGT
ATACCCCCCACTTTCTAGAAATTTAGCTCATATAGGCCCATCAGTTGATCTTGCCATTTTCTCTCTTC
ACTTAGCTGGAATCTCATCTATTTTAGGAGCTATTAATTTTATTACTACTATTATTAACATACGATGA
GAAGGAATATTAATAGAACGACTTCCTTTATTTGTATGATCTGTTTTTATTACTGCAGTTTTACTACT
TTTATCTCTACCTGTTCTTGCAGGAGCTATTACTATACTTCTTACTGACCGTAATTTCAATACCACTT
TTTTTGATCCTAGAGGAGGA
>maya
CGAACAGAATTAGGTCAACCAGGATCTCTTCTTAATGATGATCAATTATATAATGTAATTGTAACAGC
ACATGCATTTGTAATAATTTTTTTTTTAGTTATACCTGTTATAATTGGAGGATTTGGAAATTGACTAG
TACCTTTAATATTAGGAGCTCCAGATATAGCATTTCCGCGAATAAATAATATAAGTTTCTGACTTTTA
CCACCTTCTCTTACCCTATTATTATCTTCAGCTGCAGTAGAAAGAGGTGTAGGTACTGGATGAACTGT
ATATCCTCCCCTTTCAAGAAATTTAGCCCATATAGGTCCTTCCGTTGACCTAGCTATTTTTTCTCTTC
ACCTAGCCGGTATTTCATCTACTCTTGGAGCCATTAATTTTATTACAACTATTATTAATATACGATGA
GAAGGAATACTTATAGAACGACTTCCATTATTTGTATGAGCAGTTTTTATTACCGCAATCTTATTATT
ACTTTCTTTACCAGTCCTTGCTGGAGCAATTACAATACTCTTAACAGATCGAAATTTCAATACCACCT
TTTTTGATCCTAGAGGAGGA
>maya2
CGAACAGAATTAGGTCAACCAGGATCTCTTCTTAATGATGATCAATTATATAATGTAATTGTAACAGC
ACATGCATTTGTAATAATTTTTTTTTTAGTTATACCTGTTATAATTGGAGGATTTGGAAATTGACTAG
TACCTTTAATATTAGGAGCTCCAGATATAGCATTTCCGCGAATAAATAATATAAGTTTCTGACTTTTA
CCACCTTCTCTTACCCTATTATTATCTTCAGCTGCAGTAGAAAGAGGTGTAGGTACTGGATGAACTGT
ATATCCTCCCCTTTCAAGAAATTTAGCCCATATAGGTCCTTCCGTTGACCTAGCTATTTTTTCTCTTC
ACCTAGCCGGTATTTCATCTATTCTTGGAGCCATTAATTTTATTACAACTATTATTAATATACGATGA
GAAGGAATACTTATAGAACGACTTCCATTATTTGTATGAGCAGTTTTTATTACCGCAATCTTATTATT
ACTTTCTTTACCAGTCCTTGCTGGAGCAATTACAATACTCTTAACAGATCGAAATTTCAATACCACCT
TTTTTGATCCTAGAGGAGGA
>mercatoris
CGAACAGAATTAGGCCAACCAGGATCTTTATTAAATGATGATCAATTATATAATGTAATTGTAACCGC
TCATGCTTTTGTTATAATTTTTTTTTTGGTTATACCAATTATAATTGGAGGATTTGGAAACTGACTAG
TCCCCTTAATATTAGGAGCACCAGATATAGCTTTTCCTCGAATAAACAATATAAGATTTTGATTATTA
CCTCCCTCTTTAACATTACTACTTACTTCAGCTGCTGTTGAAAGAGGTGTAGGAACTGGATGAACTGT
ATACCCTCCTCTTTCTAGAAACTTAGCTCATATAGGCCCTTCAGTAGATCTTGCTATTTTTTCTCTTC
ACTTAGCTGGAATCTCTTCTATTTTAGGAGCTATTAATTTTATCACCACCATTATTAATATACGCTGA
GAAGGAATATTAATAGAACGACTTCCTTTATTTGTTTGATCAGTTTTTATTACAGCTGTATTATTACT
ATTATCCCTTCCTGTTCTAGCTGGAGCTATTACTATACTTCTTACCGATCGTAATTTTAATACTACTT
TTTTTGATCCAAGAGGAGGA

>mimicus
CGAACAGAATTAGGTCAACCAGGATCTCTTCTTAATGATGATCAATTATATAATGTTATTGTAACAGC
CCATGCTTTCGTAATAATTTTTTTCTTAGTTATACCTGTTATAATTGGAGGATTTGGTAATTGATTAG
TACCATTAATATTAGGAGCCCCTGATATAGCATTCCCCCGAATAAATAATATAAGATTTTGATTACTT
CCACCTTCACTTACACTGCTTTTATCATCAGCTGCAGTTGAAAGAGGTGTAGGAACTGGATGAACTGT
ATATCCTCCACTCTCAAGAAATTTAGCTCATATAGGACCATCAGTTGATCTTGCTATTTTTTCCCTAC
ATCTAGCAGGAATTTCATCAATTCTTGGAGCTATTAATTTTATTACCACTATTATTAATATACGATGA
GAAGGAATACTAATAGAACGACTCCCCTTATTTGTTTGATCTGTATTTATTACAGCAATTTTATTAAT
ATTATCACTACCTGTTTTAGCTGGTGCAATTACTATATTATTAACCGACCGAAATTTTAACACTACAT
TCTTTGATCCAAGAGGTGGG
>mimus2
CGAACAGAATTAGGTCAACCAGGATCTCTCCTAAATGATGATCAACTATATAACGTAATTGTTACAGC
ACATGCATTTGTAATAATTTTTTTTCTTGTTATACCTGTTATAATTGGAGGATTTGGTAATTGATTAG
TTCCCTTAATACTGGGAGCTCCAGACATAGCATTCCCACGAATAAATAATATAAGTTTTTGACTTTTA
CCACCTTCTCTTACCCTATTATTATCTTCAGCTGCAGTAGAAAGAGGTGCAGGTACTGGATGAACTGT
ATATCCCCCTCTTTCAAGAAATTTAGCTCATATAGGCCCTTCCGTTGATCTAGCTATTTTTTCTCTTC
ATTTAGCTGGTATTTCATCTATTCTTGGAGCCATTAACTTCATTACAACTATTGTCAATATACGATGA
GAAGGAATATTAATAGAACGACTTCCATTATTTGTATGATCAGTATTTATTACCGCAATTTTATTACT
TCTTTCCTTACCAGTCCTTGCTGGAGCAATTACAATACTCTTAACAGATCGAAATTTTAATACCACTT
TTTTTGACCCAAGGGGAGGA
>mimus1
CGAACAGAATTAGGTCAACCAGGATCTCTCCTAAATGATGATCAACTATATAACGTAATTGTTACAGC
ACATGCATTTGTAATAATTTTTTTTCTTGTTATACCTGTTATAATTGGAGGATTTGGTAATTGATTAG
TTCCCTTAATACTGGGAGCTCCAGATATAGCATTCCCACGAATAAATAATATAAGTTTTTGACTTTTA
CCACCTTCTCTTACCCTATTATTATCTTCAGCTGCAGTAGAAAGAGGTGCAGGTACTGGATGAACTGT
ATATCCCCCTCTTTCAAGAAATTTAGCTCATATAGGCCCTTCCGTTGATCTAGCTATTTTTTCTCTTC
ATTTAGCTGGTATTTCATCTATTCTTGGAGCCATTAACTTCATTACAACTATTGTCAATATACGATGA
GAAGGAATATTAATAGAACGACTTCCATTATTTGTATGATCAGTATTTATTACCGCAATTTTATTACT
TCTTTCCTTACCAGTCCTTGCTGGAGCAATTACAATACTCTTAACAGATCGAAATTTTAATACCACTT
TTTTTGACCCAAGGGGAGGA
>oculifer
CGAACAGAGTTAGGTCAACCAGGATCTCTCCTCAATGATGATCAATTATATAATGTAATCGTTACAGC
ACATGCATTTGTAATAATTTTTTTTTTAGTTATACCCGTTATAATTGGAGGATTTGGAAACTGATTAG
TTCCTTTAATATTAGGAGCTCCAGATATAGCATTCCCACGAATAAATAATATAAGTTTTTGACTTTTA
CCACCTTCTCTTACCCTATTACTATCTTCAGCTGCAGTAGAAAGAGGTGTAGGAACTGGATGAACTGT
ATATCCACCTCTCTCAAGTAATTTAGCCCACATAGGTCCTTCCGTTGACCTAGCTATTTTTTCTCTCC
ACCTAGCCGGTATTTCATCTATTCTTGGAGCCATTAACTTTATTACAACTATTATCAATATACGATGA
GAAGGAATATTAATAGAACGACTTCCATTATTTGTATGATCAGTATTTATTACTGCAGTTTTATTACT
ACTTTCTTTACCAGTCCTTGCTGGAGCAATTACAATACTTTTAACCGATCGAAATTTCAACACCACTT
TTTTTGATCCTAGAGGAGGA
>ornatus
CGTACTGAACTAGGCCAACCAGGCTCACTCCTCAATGACGATCAACTTTACAACGTAATTGTAACTGC
ACATGCATTTGTAATAATTTTTTTTTTAGTTATACCCGTAATAATTGGAGGATTTGGAAATTGATTAG
TCCCTTTAATATTAGGAGCCCCAGATATAGCATTTCCCCGAATAAATAATATAAGTTTCTGACTTCTA
CCTCCTTCCTTAACCTTATTATTAACTTCAGCAGCTGTTGAAAGAGGAGTAGGAACAGGTTGAACTGT
TTACCCTCCTCTATCAAGAAATCTTGCTCATACAGGCCCATCTGTAGATTTAGCAATTTTTTCACTTC
ATTTAGCTGGAATTTCATCCATCTTAGGAGCTATTAATTTTATTACTACAATTATTAATATACGATGA
GAAGGAATACAAATAGAACGTCTTCCTTTATTCGTTTGATCTGTATTTATTACAGCAATTCTTTTACT
TTTATCCTTACCTGTCCTCGCCGGAGCCATTACTATATTACTAACTGACCGAAATTTCAATACTACAT
TTTTTGACCCAAGAGGAGGA
>tetricus1
CGAACAGAACTAGGACAACCAGGGTCCCTCCTAAATGATGATCAACTATATAATGTAATTGTTACAGC
TCACGCATTTGTTATAATTTTCTTTCTTGTTATACCAGTTATAATTGGAGGATTTGGAAACTGATTAG
TTCCTTTAATACTAGGAGCACCAGATATAGCATTCCCACGAATAAATAATATAAGCTTCTGACTCCTA

CCCCCTTCTCTAACTCTTCTTCTTTCATCTGCAGCAGTCGAAAGAGGTGCAGGTACCGGATGAACCGT
TTATCCACCTCTTTCTAGAAATTTAGCTCATATAGGACCTTCTGTTGATCTAGCAATTTTCTCACTTC
ACTTAGCAGGAATTTCATCAATTCTTGGAGCCATCAACTTTATTACAACTATTATTAATATACGATGA
GAAGGTATATTAATAGAACGACTTCCACTATTTGTATGATCTGTCTTTATTACCGCAATTTTACTACT
ACTATCATTACCAGTACTCGCTGGAGCAATTACTATACTTTTAACTGACCGAAATTTTAATACAACTT
TTTTTGATCCAAGTGGAGGA
>tetricus2
CGAACAGAACTAGGACAACCAGGGTCCCTCCTAAATGATGATCAACTATATAATGTAATTGTTACAGC
TCACGCATTTGTTATAATTTTCTTTCTTGTTATACCAGTTATAATTGGAGGATTTGGAAACTGATTAG
TTCCTTTAATACTAGGAGCACCAGATATAGCATTCCCACGAATAAATAATATAAGCTTCTGACTCCTA
CCCCCTTCTCTTACTCTTCTTCTTTCATCTGCAGCAGTCGAAAGAGGTGCGGGTACCGGATGAACCGT
TTATCCACCTCTTTCAAGAAATTTAGCTCATATAGGACCTTCTGTTGATCTAGCCATTTTCTCACTTC
ACTTAGCAGGAATTTCATCAATTCTTGGAGCCATCAACTTTATTACAACTATTATTAATATACGATGA
GAAGGTATATTAATAGAACGACTTCCACTATTCGTATGATCTGTCTTTATTACCGCAATTTTACTACT
ACTATCATTACCAGTACTCGCTGGAGCAATTACTATACTTTTAACTGACCGAAATTTTAATACTACAT
TTTTTGATCCAAGTGGAGGA
>vulgaris1
CGAACAGAACTAGGTCAACCAGGATCCCTCCTAAATGATGATCAATTATATAATGTAATTGTTACAGC
TCACGCATTTGTTATAATTTTTTTCCTTGTTATACCAGTTATAATCGGAGGATTTGGAAACTGATTAG
TTCCTTTAATACTAGGAGCACCAGATATAGCATTCCCACGAATAAATAATATAAGCTTCTGACTCTTA
CCTCCTTCTCTTACTCTTCTCCTTTCATCTGCAGCAGTTGAAAGAGGCGCAGGTACCGGATGAACCGT
TTACCCGCCTCTTTCTAGAAATTTAGCTCATATAGGACCTTCTGTTGATCTAGCCATTTTCTCACTTC
ACTTAGCAGGTATTTCATCAATCCTTGGAGCCATCAACTTTATTACAACTATTATTAATATACGATGA
GAAGGTATATTAATAGAACGACTTCCACTATTTGTATGATCTGTATTTATTACCGCAATTTTACTATT
ACTATCATTACCTGTACTCGCTGGAGCAATTACTATACTTTTAACTGACCGAAATTTTAATACTACTT
TTTTTGATCCTAGTGGAGGA
>vulgaris2
CGAACAGAACTAGGACAACCAGGATCCCTCTTAAATGACGATCAACTATATAATGTAATTGTTACAGC
TCACGCATTTGTTATAATTTTTTTTCTTGTTATACCAGTTATAATTGGAGGATTCGGAAACTGATTAG
TTCCTTTAATACTAGGAGCACCAGATATAGCATTCCCACGAATAAATAACATAAGCTTTTGACTCTTA
CCCCCTTCTCTCACTCTTCTCCTTTCATCTGCAGCAGTTGAAAGAGGTGCAGGTACCGGGTGAACCGT
TTACCCTCCTCTTTCAAGAAATTTAGCTCATATAGGACCTTCTGTTGATCTAGCCATTTTCTCACTTC
ACTTAGCAGGTATTTCATCAATTCTTGGAGCCATCAACTTTATTACAACTATTATTAATATACGATGA
GAAGGTATATTAATAGAACGACTCCCACTATTTGTATGATCTGTCTTTATTACCGCAATTTTACTATT
ACTATCATTACCAGTACTTGCTGGAGCAATTACTATACTTTTAACTGATCGAAATTTTAATACCACAT
TTTTTGATCCAAGAGGAGGA
>vulgaris3
CGAACAGAACTAGGACAACCAGGATCCCTCTTAAATGACGATCAACTATATAATGTAATTGTTACAGC
TCACGCATTTGTTATAATTTTTTTTCTTGTTATACCAGTTATAATTGGAGGATTCGGAAACTGATTAG
TTCCTTTAATACTAGGAGCACCAGATATAGCATTCCCACGAATAAATAACATAAGCTTTTGACTCTTA
CCCCCTTCTCTCACTCTTCTCCTTTCATCTGCAGCAGTTGAAAGAGGTGCAGGTACCGGGTGAACCGT
TTACCCTCCTCTTTCAAGAAATTTAGCTCATATAGGACCTTCTGTTGATCTAGCCATTTTCTCACTTC
ACTTAGCAGGTATTTCATCAATTCTTGGAGCCATCAACTTTATTACAACTATTATTAATATACGATGA
GAAGGTATATTAATAGAACGACTCCCACTATTTGTATGATCTGTCTTTATTACCGCAATTTTACTATT
ACTATCATTACCAGTACTTGCTGGAGCAATTACTATACTTTTAACTGATCGAAATTTTAATACCACAT
TTTTTGATCCAAGAGGAGGA
>vulgaris4
CGAACAGAACTAGGACAACCAGGATCCCTCTTAAATGACGATCAACTATATAATGTAATTGTTACAGC
TCACGCATTTGTTATAATTTTTTTTCTTGTTATACCAGTTATAATTGGAGGATTCGGAAACTGATTAG
TTCCTTTAATACTAGGAGCACCAGATATAGCATTCCCACGAATAAATAACATAAGCTTTTGACTCTTA
CCCCCTTCTCTCACTCTTCTCCTTTCATCTGCAGCAGTTGAAAGAGGTGCAGGTACAGGATGAACCGT
TTACCCACCTCTTTCAAGAAATTTAGCTCATATAGGACCTTCTGTTGATCTAGCCATTTTCTCACTTC
ACTTAGCAGGTATTTCATCAATTCTTGGAGCCATCAACTTTATTACAACTATTATTAATATACGATGA
GAAGGTATATTAATAGAACGACTTCCACTATTTGTGTGATCTGTCTTTATTACCGCAATTTTACTATT

ACTATCATTACCAGTACTTGCTGGAGCAATTACTATACTTTTAACTGATCGAAATTTTAATACCACAT
TTTTTGATCCAAGAGGAGGA
>muslonlongi
CGAACAGAACTAGGACAACCTGGATCTTTATTAAACGATGATCAACTTTATAACGTCATTGTTACCGC
CCACGCTTTTGTAATAATTTTCTTTTTAGTTATACCCGTAATAATCGGAGGGTTTGGAAACTGATTAG
TCCCCTTAATATTGGGAGCACCAGATATAGCATTCCCACGAATAAATAATATAAGATTTTGATTACTT
CCCCCATCTTTAACCCTTCTCTTGACCTCAGCAGCAGTAGAAAGGGGAGCAGGAACAGGTTGAACTGT
TTATCCTCCATTATCAAGAAATTTAGCCCACATGGGACCATCTGTAGATCTAGCAATTTTTTCTCTTC
ACTTAGCAGGTATCTCCTCCATTTTAGGAGCCATTAATTTTATCACAACCATTATCAACATACGATGA
GAAGGTATACAAATAGAACGTCTTCCTTTATTTGTATGATCAGTTCTAATTACAGCAATCCTACTTCT
ATTATCTTTACCAGTATTAGCAGGTGCAATTACTATACTTCTCACTGACCGTAATTTCAACACAACTT
TTTTTGATCCAAGAGGAGGA
>musyaquinae
CGAACAGAACTAGGACAACCTGGATCTTTATTAAATGATGACCAACTTTATAATGTTATTGTCACTGC
CCACGCTTTTGTAATAATTTTTTTCTTAGTTATACCCGTAATAATTGGAGGATTTGGAAACTGATTAG
TTCCTTTAATATTAGGAGCACCAGATATAGCATTTCCACGAATAAATAATATAAGATTTTGATTACTC
CCTCCATCTTTAACCCTTCTCTTAACCTCAGCAGCAGTAGAAAGAGGAGCAGGAACAGGTTGAACCGT
TTACCCTCCATTATCTAGAAATTTAGCTCATATAGGACCATCTGTAGATCTAGCAATTTTCTCTCTTC
ACTTAGCGGGCATCTCCTCTATTTTAGGAGCAATTAATTTTATTACAACCATTATTAATATACGATGA
GAAGGAATACAAATAGAACGCCTTCCTTTATTTGTATGATCAGTTCTAATCACAGCAATCCTACTTCT
TCTATCCTTACCAGTATTAGCAGGTGCAATTACTATACTTCTCACTGACCGTAATTTTAATACAACCT
TTTTTGATCCTAGTGGAGGA
>musjanuari
CGAACAGAACTAGGACAACCTGGTTCCCTACTAAATGATGATCAACTCTATAACGTCATTGTTACTGC
TCACGCTTTTGTAATAATTTTCTTCTTAGTTATACCCGTAATAATTGGAGGTTTTGGTAATTGATTAG
TTCCCTTAATATTAGGAGCTCCAGACATAGCATTCCCACGAATAAATAACATAAGATTTTGATTACTT
CCCCCATCTTTAACCCTCTTATTAACCTCAGCAGCAGTAGAAAGAGGAGCAGGAACAGGTTGAACTGT
TTATCCTCCATTATCTAGAAATTTAGCTCACATAGGACCATCTGTGGATCTAGCAATTTTTTCCCTTC
ACTTAGCAGGTATTTCCTCTATTTTAGGAGCCATTAATTTCATTACAACTATTATTAATATACGATGA
GAAGGAATACAAATAGAACGTCTTCCTTTATTTGTATGATCAGTTTTAATTACAGCAATCTTACTTCT
ATTATCTTTACCAGTATTAGCAGGTGCAATTACTATACTTCTCACTGACCGTAACTTTAATACAACCT
TTTTTGACCCAAGTGGAGGA
>musakambei
CGAACAGAACTAGGACAACCTGGATCTTTATTAAACGATGATCAACTTTATAACGTCATTGTTACCGC
CCATGCTTTTGTAATAATTTTCTTTTTAGTTATACCCGTAATAATCGGAGGATTTGGAAACTGATTAG
TCCCCTTAATGTTGGGAGCACCAGATATAGCATTCCCACGAATAAATAATATAAGATTTTGATTACTT
CCCCCATCTTTAACCCTTCTCTTAACCTCAGCAGCAGTAGAAAGAGGAGCAGGAACAGGTTGAACTGT
TTATCCTCCATTATCAAGAAATTTAGCCCACATGGGACCATCTGTAGATCTAGCAATTTTTTCTCTTC
ACTTAGCAGGTATCTCCTCCATTTTAGGAGCCATTAATTTTATCACAACCATTATCAACATACGATGA
GAAGGTATACAAATAGAACGTCTTCCTTTATTTGTATGATCAGTTCTAATTACAGCAATCCTACTTCT
ATTATCTTTACCAGTATTAGCAGGTGCAATTACTATACTTCTCACTGACCGTAATTTCAACACAACTT
TTTTTGATCCAAGAGGAGGA
>whitev
CGAACAGAACTAGGCCAACCAGGATCTCTATTAAATGATGACCAATTATATAATGTAATTGTAACAGC
CCATGCTTTCGTAATAATTTTTTTTCTAGTTATACCTGTAATAATTGGAGGATTTGGAAATTGGTTAG
TACCTTTAATATTAGGAGCCCCTGATATAGCTTTTCCTCGAATAAATAATATAAGATTTTGATTACTT
CCCCCCTCATTAACACTACTTTTATCTTCAGCTGCAGTTGAAAGAGGTGTAGGAACCGGATGAACTGT
ATATCCCCCACTCTCAAGAAATTTAGCTCATATAGGACCCTCAGTTGATCTTGCTATTTTTTCTCTCC
ATTTAGCTGGTATTTCATCAATTCTTGGAGCTATTAATTTTATTACTACTATTATTAATATACGATGA
GAAGGAATATTAATAGAACGCCTTCCTTTATTTGTTTGATCTGTTTTTATTACAGCAGTCCTACTACT
TTTATCACTCCCAGTTTTAGCGGGTGCAATTACTATATTATTAACAGATCGAAACTTCAACACTACAT
TTTTTGATCCAAGAGGTGGA
