# Supplementary material for: A biogeographic framework of octopod species diversification: the role of the Isthmus of Panama
Source: PeerJ. 2020 Mar 27;8:e8691. doi: 10.7717/peerj.8691 (PMC7104719; doi:10.7717/peerj.8691)
Supplement: Supplemental Information 5 [file peerj-08-8691-s005.pdf]

>hummelinck1
CTTGGTACAGCGGTTGGGCGATTGAACGGAAAGAGGGACGGCAAAACCCTCTTCAATGCCTTGGATTC
TATTATTCCCCCGCAGAGACCAACGGAGAGACCACTGAGATTGCCTCTGCAAGATGTCTATAAGATAG
GTGGTATTGGTACCGTCCCTGTCGGCAGAGTCGAGACCGGTATCTTGAAGCCTGGTATCGTCGTAACA
TTTGCACCTGCCAATGTATCCACTGAGGTTAGTTTTTTGCTTAGGTGAAGTCTGTAGAGATGCATCAC
GAATCACTTGTAGAGGCTCTGCCAGGAGACAACGTTGGCTTTAACGTGAAGAACGTATCTGTGAAAGA
TCTTCGTCGTGGGTATGTGGCTGGAGACACCAAGTGCGAGCCTCCTAAGGAAACCAAGTCCTTTGAGG
CTCAGGTATGTCGTGAATGTATTTAATTTTTCT
>maya
CTTGGTACAGCGGTTGGTCGATTGAACGGAAAGAGGGACGGCAAAACCCTCTTCAATGCCTTGGATTC
TATTATTCCCCCGCAGAGACCAACGGAGAGACCACTGAGATTGCCTCTGCAAGATGTCTATAAGATAG
GTGGTATTGGTACCGTCCCTGTCGGCAGAGTCGAGACCGGTATCTTGAAGCCTGGTATCGTCGTAACA
TTTGCACCTGCCAATGTATCCACTGAGGTTAGTTTTTTGCTTAGGTGAAGTCTGTAGAGATGCATCAC
GAATCACTTGTAGAGGCTCTGCCAGGAGACAACGTTGGCTTTAACGTGAAGAACGTATCTGTGAAAGA
TCTTCGTCGTGGGTATGTGGCTGGAGACAGCAGGGTCGATCCTCCTAAGGAAACCAAGTCCTTTGAGG
CTCAGGTATGTCGTGAATGTATTTAATTTTTCT
>maya2
CTTGGTACAGCGGTTGGTCGATTGAACGGAAAGAGGGACGGCAAAACCCTCTTCAATGCCTTGGATTC
TATTATTCCCCCGCAGAGACCAACGGAGAGACCACTGAGATTGCCTCTGCAAGATGTCTATAAGATAG
GTGGTATTGGTACCGTCCCTGTCGGCAGAGTCGAGACCGGTATCTTGAAGCCTGGTATCGTCGTAACA
TTTGCACCTGCCAATGTATCCACTGAGGTTAGTTTTTTGCTTAGGTGAAGTCTGTAGAGATGCATCAC
GAATCACTTGTAGAGGCTCTGCCAGGAGACAACGTTGGCTTTAACGTGAAGAACGTATCTGTGAAAGA
TCTTCGTCGTGGGTATGTGGCTGGAGACAGCAGGGTCGATCCTCCTAAGGAAACCAAGTCCTTTGAGG
CTCAGGTATGTCGTGAATGTATTTAATTTTTCT
>bimaculoides1
CTTGGTACAAAGGATGGTCGATAGAGAGGAAAGAGGGGCGGCCAAACCCTCTTCAATGCCTTGGATTC
TATTATTCCCCCGCAGAGACCAACGGAGAGACCATTGAGATTGCCCCTGCAAGATGTCTACAAGATAG
GTGGTATTGGTACCGTCCCCGTCGGCAGAGTCGAGACCGGTATCTTGAAGCCTGGTATCGTCGTAACA
TTTGCACCTGCCAATATATCCACTGAGGTTAGTTTTTTGCTTAGGTGAAGTCTGTAGAAATGCATCAC
GAGTCACTTATAGAAGCTCTGCCAGGAGATAACGTTGGTTTTAACGTTAAGAACGTATCGGTTAAGGA
TCTTCGTCGTGGATATGTGGCTGGAGACAGCAAGAACGATCCCCCTAGGGAAACGAAGAACTTTGAGG
CTCAGGTTTGTCGTGAATGTATTTAATTTTTCT
>vulgaris4
CTTGGTACGAAGGCTGGGAAATTGAGAGGAAAGAGGGAGGGCAATACCCTCTTCAATGCCTTGGATTC
TATTATTCCGCCGCAGAGACCAACGGAGAGACCATTGAGATTGCCCCTGCAGGATGTTTACAAGATAG
GTGGTATTGGTACCGTCCCTGTTGGCAGAGTCGAGACCGGTGTCTTGAAGCCTGGTACCGTCGTGACA
TTTGCACCTGCCATGGTATCCACTGAGGTTAGTTTTTTGCTTAGGTGAAGTCTGTAGAGATGCACCAC
GAGTCACTTCCAGAAGCTAACCCAGGAGACAACGTTGGTTTTAACGTTAAGAACGTATCTGTAAAGGA
TCTTCGTCGTGGGTATGTGGCTGGTGACAGCAAGAACGACCCTCCTAAGGAAACGAAGTGCTTTGATG
CCCAGGTATGTCGTGATTGTGTTTAATTTTTCT
>vulgaris2
CTTGGTACGATGGCTGGGAAATTGAGAGGAAAGAGGGAGGGCAGTACCCTCTTCAATGCCTTGGATTC
TATTATTCCGCCGCAGAGACCAACGGAGAGACCATTGAGATTGCCCCTGCAGGATGTCTACAAGATAG
GTGGTATTGGTACCGTCCCTGTTGGCAGAGTCGAGACCGGTGTCTTGAAGCCTGGTACCGTCGTGACA
TTTGCACCTGCCATGGTATCCACTGAGGTTAGTTTTTTGCTTAGGTGAAGTCTGTAGAGATGCACCAC
GAGTCACTTCCAGAAGCTAACCCAGGAGACAACGTTGGTTTTAACGTGAAGAACGTATCTGTAAAGGA
TCTTCGTCGTGGGTATGTGGCTGGTGACAGCAAGAACGACCCTCCTAAGGAAACGAAGTGCTTTGATG
CCCAGGTATGTCGTGATTGTGTTTAATTTTTCT
>insularis1
CTTGGTACACCGGTTGGTCGATTGAACGGAAAGAGGGGCGGCAAAACCCTCTTCAATGCCTTGGATTC
TATTATTCCCCCGCAGAGACCAACGGAGAGACCACTGAGATTGCCTCTGCAAGATGTCTATAAGATAG
GTGGTATTGGTACCGTCCCTGTCGGCAGAGTCGAGACCGGTATCTTGAAGCCTGGTATCGTCGTAACA
TTTGCACCTGCCAATGTATCCACTGAGGTTAGTTTTTTGCTTAGGTGAAGTCTGTAGAGATGCATCAC
GAATCACTTGTAGAGGCTCTGCCAGGAGACAACGTTGGCTTTAACGTGAAGAACGTATCTGTGAAAGA

```
TCTTCGTCGTGGGTATGTGGCTGGAGACAGCAAGAGCGATCCACCTAAGGAAACCAAGTCCTTTGAGG
CTCAGGTATGTCGTGAATGTATTTAATTTTTCT
>insularis4
CTTGGTACACCGGTTGGTCGACTGAACGGAAAGAGGGGCGGCAAAACCCTCTTCAATGCCTTGGATTC
TATTATTCCCCCGCAGAGACCAACGGAGAGACCACTGAGATTGCCTCTGCAAGATGTCTATAAGATAG
GTGGTATTGGTACCGTCCCTGTCGGCAGAGTCGAGACCGGTATCTTGAAGCCTGGTATCGTCGTAACA
TTTGCACCTGCCAATGTATCCACTGAGGTTAGTTTTTTGCTTAGGTGAAGTCTGTAGAGATGCATCAC
GAATCACTTGTAGAGGCTCTGCCAGGAGACAACGTTGGCTTTAACGTGAAGAACGTATCTGTGAAAGA
TCTTCGTCGTGGGTATGTGGCTGGAGACAGCAAGAGCGATCCACCTAAGGAAACCAAGTCCTTTGAGG
CTCAGGTATGTCGTGAATGTATTTAATTTTTCT
>insularis5
CTTGGTACACCGGTTGGTCGATTGAACGGAAAGAGGGGCGGCAAAACCCTCTTCAATGCCTTGGATTC
TATTATTCCCCCGCAGAGACCAACGGAGAGACCACTGAGATTGCCTCTGCAAGATGTCTATAAGATAG
GTGGTATTGGTACCGTCCCTGTCGGCAGAGTCGAGACCGGTATCTTGAAGCCTGGTATCGTCGTAACA
TTTGCACCTGCCAATGTATCCACTGAGGTTAGTTTTTTGCTTAGGTGAAGTCTGTAGAGATGCATCAC
GAATCACTTGTAGAGGCTCTGCCAGGAGACAACGTTGGCTTTAACGTGAAGAACGTATCTGTGAAAGA
TCTTCGTCGTGGGTATGTGGCTGGAGACAGCAAGAGCGATCCACCTAAGGAAACCAAGTCCTTTGAGG
CTCAGGTATGTCGTGAATGTATTTAATTTTTCT
>vulgaris1
CTTGGTACGAAGGCTGGGAAATTGAGAGGAAAGAGGGAGGGCAATACCCTCTTCAATGCCTTGGATTC
TATTATTCCGCCGCAGAGACCAACGGAGAGACCATTGAGATTGCCCCTGCAGGATGTCTACAAGATAG
GTGGTATTGGTACCGTCCCTGTTGGCAGAGTCGAGACCGGTGTCTTGAAGCCTGGTACCGTCGTGACA
TTTGCACCTGCCATGGTATCCACTGAGGTTAGTTTTTTGCTTAGGTGAAGTCTGTAGAGATGCACCAC
GAGTCACTTCCAGAAGCTAACCCAGGAGACAACGTTGGTTTTAACGTTAAGAACGTATCTGTAAAGGA
TCTTCGTCGTGGGTATGTGGCTGGTGACAGCAAGAACGACCCTCCTAAGGAAACGAAGTGCTTTGATG
CCCAGGTATGTCGTGATTGTGTTTAATTTTTCT
>vulgaris3
CTTGGTACGATGGCTGGGAAATTGAGAGGAAAGAGGGAGGGCAGTACCCTCTTCAATGCCTTGGATTC
TATTATTCCGCCGCAGAGACCAACGGAGAGACCATTGAGATTGCCCCTGCAGGATGTCTACAAGATAG
GTGGTATTGGTACCGTCCCTGTTGGCAGAGTCGAGACCGGTGTCTTGAAGCCTGGTACCGTCGTGACA
TTTGCACCTGCCATGGTATCCACTGAGGTTAGTTTTTTGCTTAGGTGAAGTCTGTAGAGATGCACCAC
GAGTCACTTCCAGAAGCTAACCCAGGAGACAACGTTGGTTTTAACGTGAAGAACGTATCTGTAAAGGA
TCTTCGTCGTGGGTATGTGGCTGGTGACAGCAAGAACGACCCTCCTAAGGAAACGAAGTGCTTTGATG
CCCAGGTATGTCGTGATTGTGTTTAATTTTTCT
>tetricus1
CTTGGTACGAAGGCTGGGAAATTGAGAGGAAAGAGGGAGGGCAATACCCTCTTCAATGCCTTGGATTC
TATTATTCCGCCGCAGAGACCAACGGAGAGACCATTGAGATTGCCCCTGCAGGATGTCTACAAGATAG
GTGGTATTGGTACCGTCCCTGTTGGCAGAGTCGAGACCGGTGTCTTGAAGCCTGGTACCGTCGTGACA
TTTGCACCTGCCATGGTATCCACTGAGGTTAGTTTTTTGCTTAGGTGAAGTCTGTAGAGATGCACCAC
GAGTCACTTTCAGAAGCTAACCCAGGAGACAATGTTGGTTTTAACGTTAAGAACGTATCTGTAAAGGA
TCTTCGTCGTGGGTATGTGGCTGGTGACAGCAAGAACGACCCTCCTAAGGAAACGAAGTGCTTTGATG
CCCAGGTATGTCGTGATTGTGTTTAATTTTTCT
>tetricus2
CTTGGTACGATGGCTGGGAAATTGAGAGGAAAGAGGGAGGGCAATACCCTCTTCAATGCCTTGGATTC
TATTATTCCGCCGCAGAGACCAACGGAGAGACCATTGAGATTGCCCCTGCAGGATGTCTACAAGATAG
GTGGTATTGGTACCGTCCCTGTTGGCAGAGTCGAGACCGGTGTCTTGAAGCCTGGTACCGTCGTGACA
TTTGCACCTGCCATGGTATCCACTGAGGTTAGTTTTTTGCTTAGGTGAAGTCTGTAGAGATGCACCAT
GAGTCACTTCCAGAAGCTAACCCAGGAGACAACGTTGGTTTTAACGTTAAGAACGTATCTGTAAAGGA
TCTTCGTCGTGGGTATGTGGCTGGTGACAGCAAGAACGACCCTCCTAAGGAAACGAAGTGCTTTGATG
CCCAGGTATGTCGTGATTGTGTTTAATTTTTCT
```
