# Supplementary material for: A biogeographic framework of octopod species diversification: the role of the Isthmus of Panama
Source: PeerJ. 2020 Mar 27;8:e8691. doi: 10.7717/peerj.8691 (PMC7104719; doi:10.7717/peerj.8691)
Supplement: Supplemental Information 6 [file peerj-08-8691-s006.pdf]

>384
GACCTATGGCGGCATCCAAGAAAATGTCCCATAGAAGAGCTTTCCTCATGATTATCTTTGTGTGGATG
TGGTCCATTGTATGGGCTGTTGGTCCCGTTTTCAACTGGGGAGCCTACGTCCCTGAAGGTATCCTAAC
ATCTTGCTCTTTTGATTACCTGTCTACTGATTCTAGCACCAAATCTTTCATCTTGTGCATGTACTTCT
GCGGCTTCATGCTGCCCGTAGTTATCATCGCTTTCTGTTACTTCAACATTGTCATGTCTGTATCCAAC
CACGAAAAGGAAATGGCTGCCATGGCTAAGAGGTTGAACGCCAAAGAATTGCGTAAGGCACAGGCCGG
CCAGAGCGCTGAAATGAAACTTGCTAAAATTTCAATGGTAATTATTACCCAATTTATGCTTTCCTGGT
CTCCATACGCCATCATCGCTCTTCTTGCACAGTTTGGGCCAGCTGAATGGGTTACCCCATACGCAGCC
GAGTTGCCTGTCCTGTTTGCTAAAGCTTCAGCTATCCACAACCCAATTGTCTACTCTGTTTC
>385
GACCTATGGCGGCATCCAAGAAAATGTCCCATAGAAGAGCTTTCCTCATGATTATCTTTGTGTGGATG
TGGTCCATTGTATGGGCTGTTGGTCCCGTTTTCAACTGGGGAGCCTACGTCCCTGAAGGTATCCTAAC
ATCTTGCTCTTTTGATTACCTGTCTACTGATTCTAGCACCAAATCTTTCATCTTGTGCATGTACTTCT
GCGGCTTCATGCTGCCCGTAGTTATCATCGCTTTCTGTTACTTCAACATTGTCATGTCTGTATCCAAC
CACGAAAAGGAAATGGCTGCCATGGCTAAGAGGTTGAACGCCAAAGAATTGCGTAAGGCACAGGCCGG
CCAGAGCGCTGAAATGAAACTTGCTAAAATTTCAATGGTAATTATTACCCAATTTATGCTTTCCTGGT
CTCCATACGCCATCATCGCTCTTCTTGCACAGTTTGGGCCAGCTGAATGGGTTACCCCATACGCAGCC
GAGTTGCCTGTCCTGTTTGCTAAAGCTTCAGCTATCCACAACCCAATTGTCTACTCTGTTTC
>386
GACCTATGGCGGCATCCAAGAAAATGTCCCATAGAAGAGCTTTCCTCATGATTATCTTTGTGTGGATG
TGGTCCATTGTATGGGCTGTTGGTCCCGTTTTCAACTGGGGAGCCTACGTCCCTGAAGGTATCCTAAC
ATCTTGCTCTTTTGATTACCTGTCTACTGATTCTAGCACCAAATCTTTCATCTTGTGCATGTACTTCT
GCGGCTTCATGCTGCCCGTAGTTATCATCGCTTTCTGTTACTTCAACATTGTCATGTCTGTATCCAAC
CACGAAAAGGAAATGGCTGCCATGGCTAAGAGGTTGAACGCCAAAGAATTGCGTAAGGCACAGGCCGG
CCAGAGCGCTGAAATGAAACTTGCTAAAATTTCAATGGTAATTATTACCCAATTTATGCTTTCCTGGT
CTCCATACGCCATCATCGCTCTTCTTGCACAGTTTGGGCCAGCTGAATGGGTTACCCCATACGCAGCC
GAGTTGCCTGTCCTGTTTGCTAAAGCTTCAGCTATCCACAACCCAATTGTCTACTCTGTTTC
>bimaculatus
GACCTATGGCGGCATCCAAGAAAATGTCCCATAGAAGAGCTTTCCTCATGATTATCTTTGTGTGGATA
TGGTCCATTGTTTGGTCTGTCGGCCCCGTCTTCAACTGGGGAGCATACGTCCCTGAAGGTATTCTTAC
ATCTTGCTCTTTTGATTACCTGTCTACTGATTCTAGTACCAGATCTTTCATCTTGTGCATGTACTTCT
GTGGTTTCATGCTGCCCATAATTATCATCGCTTTCTGTTACTTCAACATTGTCATGTCTGTATCCAAC
CATGAAAAGGAAATGGCTGCCATGGCTAAGAGGTTGAACGCCAAAGAATTGCGTAAGGCCCAGGCTGG
CCAGAGCGCTGAAATGAAACTCGCCAAAATCTCAATGGTAATTATTACCCAATTCATGCTCTCCTGGT
CTCCATACGCCATCATCGCTCTTCTTGCACAGTTTGGGCCAGCTGAATGGGTTACTCCTTATGCAGCT
GAATTGCCTGTCCTGTTTGCTAAAGCTTCAGCTATCCACAACCCAATTGTCTACTCTGTTTC
>bimaculoides1
GACCTATGGCGGCATCCAAGAAAATGTCCCATAGAAGAGCTTTCCTCATGATTATCTTTGTGTGGATA
TGGTCCATTGTTTGGTCTGTCGGCCCCGTCTTCAACTGGGGAGCATACGTCCCTGAAGGTATTCTTAC
ATCTTGCTCTTTTGATTACCTGTCTACTGATTCTAGTACCAGATCTTTCATCTTGTGCATGTACTTCT
GTGGTTTCATGCTGCCCATAATTATCATCGCTTTCTGTTACTTCAACATTGTCATGTCTGTATCCAAC
CATGAAAAGGAAATGGCTGCCATGGCTAAGAGGTTGAACGCCAAAGAATTGCGTAAGGCCCAGGCTGG
CCAGAGCGCTGAAATGAAACTCGCCAAAATCTCAATGGTAATTATTACCCAATTCATGCTCTCCTGGT
CTCCATACGCCATCATCGCTCTTCTTGCACAGTTTGGGCCAGCTGAATGGGTTACTCCTTATGCAGCT
GAATTGCCTGTCCTGTTTGCTAAAGCTTCAGCTATCCACAACCCAATTGTCTACTCTGTTTC
>bimaculoides2
GACCTATGGCGGCATCCAAGAAAATGTCCCATAGAAGAGCTTTCCTCATGATTATCTTTGTGTGGATA
TGGTCCATTGTTTGGTCTGTCGGCCCCGTCTTCAACTGGGGAGCATACGTCCCTGAAGGTATTCTTAC
ATCTTGCTCTTTTGATTACCTGTCTACTGATTCTAGTACCAGATCTTTCATCTTGTGCATGTACTTCT
GTGGTTTCATGCTGCCCATAATTATCATCGCTTTCTGTTACTTCAACATTGTCATGTCTGTATCCAAC
CATGAAAAGGAAATGGCTGCCATGGCTAAGAGGTTGAACGCCAAAGAATTGCGTAAGGCCCAGGCTGG
CCAGAGCGCTGAAATGAAACTCGCCAAAATCTCAATGGTAATTATTACCCAATTCATGCTCTCCTGGT
CTCCATACGCCATCATCGCTCTTCTTGCACAGTTTGGGCCAGCTGAATGGGTTACTCCTTATGCAGCT
GAATTGCCTGTCCTGTTTGCTAAAGCTTCAGCTATCCACAACCCAATTGTCTACTCTGTTTC

```
>defilippi1
GACCTATGGCGGCATCCAAGAAAATGTCCCATAGAAGAGCTTTCCTCATGATTATCTTTGTGTGGATG
TGGTCCATTGTTTGGGCTGTTGGCCCCGTCTTCAACTGGGGAGCATACGTCCCTGAAGGTATTCTTAC
ATCTTGCTCTTTTGATTACCTGTCTACTGATCCTAGTACCAGATCTTTCATCTTGTGCATGTACTTCT
GTGGTTTCATGCTGCCCATTATTATCATCGCTTTCTGTTACTTCAACATTGTCATGTCTGTATCCAAC
CATGAAAAGGAAATGGCTGCCATGGCTAAGAGGTTGAACGCCAAAGAATTGCGTAAAGCACAGGCTGG
CCAGAGCGCTGAAATGAAACTTGCCAAAATCTCAATGGTAATTATCACCCAATTCATGCTCTCCTGGT
CTCCATACGCCGTCATTGCTCTTCTTGCCCAGTTTGGTCCAGCTGAATGGGTCACCCCATATGCAGCT
GAATTGCCTGTCCTCTTCGCTAAAGCCTCAGCTATCCACAACCCAATTGTCTACTCTGTTTC
>defilippi2
GACCTATGGCGGCATCCAAGAAAATGTCCCATAGAAGAGCTTTCCTCATGATTATCTTTGTGTGGATG
TGGTCCATTGTTTGGGCTGTTGGCCCCGTCTTCAACTGGGGAGCATACGTCCCTGAAGGTATTCTTAC
ATCTTGCTCTTTTGATTACCTGTCTACTGATCCTAGTACCAGATCTTTCATCTTGTGCATGTACTTCT
GTGGTTTCATGCTGCCCATTATTATCATCGCTTTCTGTTACTTCAACATTGTCATGTCTGTATCCAAC
CATGAAAAGGAAATGGCTGCCATGGCTAAGAGGTTGAACGCCAAAGAATTGCGTAAAGCACAGGCTGG
CCAGAGCGCTGAAATGAAGCTTGCCAAAATCTCAATGGTAATTATCACCCAATTCATGCTCTCCTGGT
CTCCATACGCCGTCATTGCTCTTCTTGCCCAGTTTGGTCCAGCTGAATGGGTCACCCCATATGCAGCT
GAATTGCCTGTCCTCTTCGCTAAAGCCTCAGCTATCCACAACCCAATTGTCTACTCTGTTTC
>digueti
GACCTATGGCGGCATCCAAGAAAATGTCCCATAGAAGAGCTTTCCTCATGATTATCTTTGTGTGGATG
TGGTCCATTATTTGGGCTGTCGGTCCCGTCTTCAACTGGGGAGCATACGTCCCTGAAGGTATTCTCAC
ATCTTGCTCTTTTGATTACCTGTCTACTGATTCTAGCACAAGATCTTTCATCTTGTGCATGTACTTCT
GTGGTTTCATGTTACCCGTAGTTATCATCGCTTTCTGTTACTTCAACATTGTCATGTCTGTATCCAAC
CATGAAAAGGAAATGGCTGCCATGGCTAAGAGGTTGAACGCCAAAGAATTGCGTAAGGCACAGGCTGG
CCAGAGCGCTGAAATGAAACTTGCCAAGATTTCAATGGTAATTATTACCCAATTCATGCTTTCCTGGT
CTCCATACGCTATCATCGCTCTTCTTGCACAGTTTGGGCCAGCTGAATGGGTTACCCCATACGCAGCT
GAATTACCTGTCCTGTTTGCTAAAGCTTCAGCTATCCACAACCCAATTGTCTACTCTGTTTC
>dofleini
GACCTATGGCAGCGTCCAAAAAAATGTCCCATAGAAGAGCTTTCCTCATGATTATCTTTGTGTGGATG
TGGTCCATTGTTTGGTCAGTCGGACCCGTCTTCAACTGGGGAGCATACGTCCCCGAAGGTATTCTCAC
ATCCTGCTCTTTCGATTACCTCTCCACTGATCCTAGTACCAGATCTTTCATCTTGTGCATGTACTTCT
GTGGTTTCATGCTGCCCATAATTATCATCGCTTTCTGTTATTTCAACATTGTCATGTCTGTATCCAAC
CACGAAAAGGAAATGGCTGCCATGGCAAAGAGGTTGAATGCCAAAGAATTGCGTAAAGCACAGGCTGG
TGCGAGCGCTGAAATGAAACTTGCCAAAATTTCAATGGTAATTATTACCCAATTCATGCTTTCCTGGT
CTCCATACGCCATCATCGCTCTTCTTGCACAGTTTGGGCCAGCTGAATGGGTTACTCCATACGCAGCC
GAATTGCCTGTACTGTTTGCTAAAGCTTCAGCTATCCACAACCCAATTGTCTACTCTGTTTC
>fitchi
GACCTATGGCGGCATCCAAGAAAATGTCCCATAGACGAGCTTTCCTCATGATTATCTTTGTGTGGATG
TGGTCCATTATTTGGGCTGTCGGTCCCGTCTTCAACTGGGGAGCATACGTCCCTGAAGGTATTCTCAC
ATCTTGCTCTTTTGATTATCTGTCTACCGATTCTAGCACAAGATCTTTCATCTTGTGCATGTACTTCT
GTGGTTTCATGCTACCCGTAGTTATCATCGCTTTCTGTTATTTCAACATTGTCATGTCTGTATCCAAC
CATGAAAAGGAAATGGCTGCCATGGCTAAGAGGTTGAACGCCAAAGAATTGCGTAAGGCACAGGCTGG
CCAGAGCGCTGAAATGAAACTTGCCAAGATTTCAATGGTAATTATTACCCAATTCATGCTTTCCTGGT
CTCCATACGCCATCATCGCTCTTCTTGCACAGTTTGGGCCAGCTGAATGGGTTACCCCATACGCAGCT
GAATTACCTGTCCTGTTTGCTAAAGCTTCAGCTATCCACAACCCAATTGTCTACTCTGTTTC
>musyaquinae
GACCTATGGCAGCGTCCAAAAAGATGTCCCATAGAAAAGCTTTCCTCATGATTATCTTTGTGTGGATG
TGGGCCATTGTTTGGGCAGTCGGACCCATCTTCAACTGGGGTGCATACGTCCCGGAAGGTATTCTCAC
ATCCTGCTCTTTTGATTACCTCTCCACTGATTCTACTACTAGATCTAACGTCTTGTGCATGTACTTTT
GTGGTTTCACGATGCCCATAGTTATCATCGCTTTCTGTTATTTCAACATTGTCATGTCTGTATCCAAC
CACGAAAAGGAGATGGCTGCCATGGCAAAGAGGTTGAATGCCAAAGAATTGCGTAAAGCACAGGCTGG
CCAGAGCTCTGAAATGAAACTTGCCAAAATTTCGATGGTAATTATTACCCAATTCTTGCTTTCCTGGT
CTCCATACGCCATCGTCGCTCTTCTTGCACAGTTTGGACCAGCTGAATGGATTACTCCATACGCAGCT
GAATTGCCTGTACTGTTTGCTAAAGCTTCAGCTATCCACAACCCAATTGTCTACTCTGTTTC
```

>muslonlongi
GACCTATGGCAGCGTCCAAAAAGATGTCCCATAGAAAAGCTTTCCTCATGATTATCTTTGTGTGGATG
TGGGCCATTGTTTGGGCAGTCGGACCCATCTTCAACTGGGGTGCATACGTCCCGGAAGGTATTCTCAC
ATCCTGCTCTTTTGATTACCTCTCCACTGATTCTACTACTAGATCTAACATCTTGTGCATGTACTTCT
GTGGTTTCATGATGCCCATAGTTATCATCGGTTTYTGTTATTTCAACATTGTCATGTCTGTATCCAAC
CACGAAAAGGAGATGGCTGCCATGGCAAAGAGGTTGAATGCCAAAGAATTGCGTAAAGCACAGGCTGG
CCAGAGCGCTGAAATGAAACTTGCCAAAATTTCGATGATAATTATTACCCAATTCTTGCTTTCCTGGT
CTCCATACGCCATCGTCGCTCTTCTTGCACAGTTTGGGCCAGCTGAATGGATTACTCCATACGCAGCT
GAATTGCCTGTACTGTTTGCTAAAGCTTCAGCTATCCACAACCCAATTGTCTACTCTGTTTC
>musakambei
GACCTATGGCAGCGTCCAAAAAGATGTCCCATAGAAAAGCTTTCCTCATGATTATCTTTGTGTGGATG
TGGGCCATTGTTTGGGCAGTCGGACCCATCTTCAACTGGGGTGCATATGTCCCGGAAGGTATTCTCAC
ATCCTGCTCTTTTGATTACCTCTCCACTGATTCTACTACTAGATCTAACATCTTGTGCATGTACTTCT
GTGGTTTCATGATGCCCATAGTTATCATCGGTTTCTGTTATTTCAACATTGTCATGTCTGTATCCAAC
CACGAAAAGGAGATGGCTGCCATGGCAAAGAGGTTGAATGCCAAAGAATTGCGTAAAGCACAGGCTGG
CCAGAGCGCTGAAATGAAACTTGCCAAAATTTCGATGATAATTATTACCCAATTCATGCTTTCCTGGT
CTCCATACGCCATCGTCGCTCTTCTTGCACAGTTTGGGCCAACTGAATGGATTACTCCATTAGCAGCT
GAATTGCCTGTACTGTTTGCTAAAGCTTCAGCTATCCACAACCCAATTGTCTACTCTGTTTC
>musjanuari
GACCTATGGCAGCGTCCAAAAAGATGTCCCATAGAAAAGCTTTCCTCATGATTATCTTTGTGTGGATG
TGGGCCATTGTTTGGGCAGTCGGACCCATCTTCAACTGGGGTGCATACGTCCCGGAAGGTATTCTCAC
ATCCTGCTCTTTTGATTACCTCTCCACTGATTCTAGTACAAGATCTTTCATCTTGTGCATGTACTTCT
GTGGTTTCACAATGCCCATAGTTATCATCGCTTTCTGTTATTTCAACATTGTCATGTCTGTATCCAAC
CACGAAAAGGAGATGGCTGCCATGGCAAAGAGGTTGAATGCCAAAGAATTGCGTAAAGCACAGGCTGG
CCAGAGCGCTGAAATGAAACTCGCCAAAATTTCGATGGTAATTATTACCCAATTCTTGGTTTCCTGGT
CTCCATACGCCATCGTCGCTCTTCTTGCACAGTTTGGGCCAGCTGAATGGGTTACTCCATACGCAGCT
GAATTGCCTGTACTGTTTGCTAAAGCTTCAGCTATCCACAACCCAATTGTCTACTCTGTTTC
>hummelinck1
GACCTATGGCGGCATCCAAGAAAATGTCCCATAGAAGAGCTTTCCTCATGATTATCTTTGTGTGGATA
TGGTCCATTGTTTGGTCTGTCGGCCCCGTCTTCAACTGGGGAGCATACGTCCCTGAAGGTATTCTTAC
ATCTTGCTCTTTTGATTACCTGTCTACTGATTCTAGTACCAGATCTTTCATCTTGTGCATGTACTTCT
GTGGTTTCATGCTGCCCGTAGTTATCATCGCTTTCTGCTACTTCAACATTGTCATGTCTGTATCCAAC
CACGAAAAGGAAATGGCTGCCATGGCTAAGAGGTTGAACGCCAAAGAATTGCGTAAGGCACAGGCTGG
CCAGAGCGCTGAAATGAAACTCGCCAAAATTTCAATGGTAATTATTACCCAATTCATGCTCTCCTGGT
CTCCATACGCCATCATCGCTCTTCTTGCACAGTTTGGGCCAGCTGAATGGGTTACTCCTTATGCAGCT
GAATTGCCTGTCCTGTTTGCTAAAGCTTCAGCTATCCACAACCCAATTGTCTACTCTGTTTC
>hummelinck3
GACCTATGGCGGCATCCAAGAAAATGTCCCATAGAAGAGCTTTCCTCATGATTATCTTTGTGTGGATA
TGGTCCATTGTTTGGTCTGTCGGCCCCGTCTTCAACTGGGGAGCATACGTCCCTGAAGGTATTCTTAC
ATCTTGCTCTTTTGATTACCTGTCTACTGATTCTAGTACCAGATCTTTCATCTTGTGCATGTACTTCT
GTGGTTTCATGCTGCCCGTAGTTATCATCGCTTTCTGCTACTTCAACATTGTCATGTCTGTATCCAAC
CACGAAAAGGAAATGGCTGCCATGGCTAAGAGGTTGAACGCCAAAGAATTGCGTAAGGCACAGGCTGG
CCAGAGCGCTGAAATGAAACTCGCCAAAATTTCAATGGTAATTATTACCCAATTCATGCTCTCCTGGT
CTCCATACGCCATCATCGCTCTTCTTGCACAGTTTGGGCCAGCTGAATGGGTTACTCCTTATGCAGCT
GAATTGCCTGTCCTGTTTGCTAAAGCTTCAGCTATCCACAACCCAATTGTCTACTCTGTTTC
>insularis1
GACCTATGGCGGCATCCAAGAAAATGTCCCATAGAAGAGCTTTCCTCATGATTATCTTTGTGTGGATA
TGGTCCATTGTTTGGGCTGTCGGCCCCGTCTTCAACTGGGGAGCATACGTCCCTGAAGGTATTCTTAC
ATCTTGCTCTTTTGATTACCTGTCTACTGATTCTAGTACCAGATCTTTCATCTTGTGCATGTACTTCT
GTGGTTTCATGCTGCCCGTAGTTATCATCGCTTTCTGTTACTTCAACATTGTCATGTCTGTATCCAAC
CACGAAAAGGAAATGGCTGCCATGGCTAAGAGGTTGAACGCCAAAGAATTGCGTAAGGCACAGGCTGG
CCAGAGCGCTGAAATGAAACTCGCCAAAATTTCAATGGTAATTATTACCCAATTCATGCTCTCCTGGT
CTCCATACGCCATCATCGCTCTTCTTGCACAGTTTGGGCCAGCTGAATGGGTTACTCCTTATGCAGCT
GAATTGCCTGTCCTGTTTGCTAAAGCTTCAGCTATCCACAACCCAATTGTCTACTCTGTTTC

>insularis4
GACCTATGGCGGCATCCAAGAAAATGTCCCATAGAAGAGCTTTCCTCATGATTATCTTTGTGTGGATA
TGGTCCATTGTTTGGGCTGTCGGCCCCGTCTTCAACTGGGGAGCATACGTCCCTGAAGGTATTCTTAC
ATCTTGCTCTTTTGATTACCTGTCTACTGATTCTAGTACCAGATCTTTCATCTTGTGCATGTACTTCT
GTGGTTTCATGCTGCCCGTAGTTATCATCGCTTTCTGTTACTTCAACATTGTCATGTCTGTATCCAAC
CACGAAAAGGAAATGGCTGCCATGGCTAAGAGGTTGAACGCCAAAGAATTGCGTAAGGCACAGGCTGG
CCAGAGCGCTGAAATGAAACTCGCCAAAATTTCAATGGTAATTATTACCCAATTCATGCTCTCCTGGT
CTCCATACGCCATCATCGCTCTTCTTGCACAGTTTGGGCCAGCTGAATGGGTTACTCCTTATGCAGCT
GAATTGCCTGTCCTGTTTGCTAAAGCTTCAGCTATCCACAACCCAATTGTCTACTCTGTTTC
>insularis5
GACCTATGGCGGCATCCAAGAAAATGTCCCATAGAAGAGCTTTCCTCATGATTATCTTTGTGTGGATA
TGGTCCATTGTTTGGGCTGTCGGCCCCGTCTTCAACTGGGGAGCATACGTCCCTGAAGGTATTCTTAC
ATCTTGCTCTTTTGATTACCTGTCTACTGATTCTAGTACCAGATCTTTCATCTTGTGCATGTACTTCT
GTGGTTTCATGCTGCCCGTAGTTATCATCGCTTTCTGTTACTTCAACATTGTCATGTCTGTATCCAAC
CACGAAAAGGAAATGGCTGCCATGGCTAAGAGGTTGAACGCCAAAGAATTGCGTAAGGCACAGGCTGG
CCAGAGCGCTGAAATGAAACTCGCCAAAATTTCAATGGTAATTATTACCCAATTCATGCTCTCCTGGT
CTCCATACGCCATCATCGCTCTTCTTGCACAGTTTGGGCCAGCTGAATGGGTTACTCCTTATGCAGCT
GAATTGCCTGTCCTGTTTGCTAAAGCTTCAGCTATCCACAACCCAATTGTCTACTCTGTTTC
>joubini3
GACCTATGGCGGCATCTAAGAAAATGTCCCATAGAAGAGCTTTCCTCATGATTATCTTTGTGTGGATG
TGGTCCATTGTTTGGGCTGTCGGTCCCGTCTTCAACTGGGGAGCATACGTCCCTGAAGGTATTCTCAC
ATCTTGCTCTTTTGATTACCTGTCTACTGATTCTAACACAAGATCTTTCATCTTGTGCATGTACTTCT
GTGGTTTCATGTTACCCGTAGTTATCATCGCTTTCTGTTACTTCAACATTGTCATGTCTGTATCCAAC
CATGAAAAGGAAATGGCTGCCATGGCTAAGAGGTTGAACGCCAAAGAATTGCGTAAGGCACAGGCTGG
CCAGAGCGCTGAAATGAAACTTGCCAAGATTTCAATGGTAATTATTACCCAATTCATGCTTTCCTGGT
CTCCATACGCCATCATCGCTCTTCTTGCACAGTTTGGGCCAGCTGAATGGGTTACCCCATACGCAGCT
GAATTACCTGTCCTGTTTGCTAAAGCTTCAGCTATCCACAACCCAATTGTCTACTCTGTTTC
>maya
GACCTATGGCGGCATCCAAGAAAATGTCCCATAGAAGAGCTTTCCTCATGATTATCTTTGTGTGGATA
TGGTCCATTGTTTGGTCTGTCGGCCCCGTCTTCAACTGGGGAGCATACGTCCCTGAAGGTATTCTTAC
ATCTTGCTCTTTTGATTACCTGTCTACTGATTCTAGTACCAGATCTTTCATCTTGTGCATGTACTTCT
GTGGTTTCATGCTGCCCGTAGTTATCATCGCTTTCTGTTACTTCAACATTGTCATGTCTGTATCCAAC
CACGAAAAGGAAATGGCTGCCATGGCTAAGAGGTTGAACGCCAAAGAATTGCGTAAGGCACAGGCTGG
CCAGAGCGCTGAAATGAAACTCGCCAAAATTTCAATGGTAATTATTACCCAATTCATGCTCTCCTGGT
CTCCATACGCCATCATCGCTCTTCTTGCACAGTTTGGGCCAGCTGAATGGGTTACTCCTTATGCAGCT
GAATTGCCTGTCCTGTTTGCTAAAGCTTCAGCTATCCACAACCCAATTGTCTACTCTGTTTC
>maya2
GACCTATGGCGGCATCCAAGAAAATGTCCCATAGAAGAGCTTTCCTCATGATTATCTTTGTGTGGATA
TGGTCCATTGTTTGGTCTGTCGGCCCCGTCTTCAACTGGGGAGCATACGTCCCTGAAGGTATTCTTAC
ATCTTGCTCTTTTGATTACCTGTCTACTGATTCTAGTACCAGATCTTTCATCTTGTGCATGTACTTCT
GTGGTTTCATGCTGCCCGTAGTTATCATCGCTTTCTGTTACTTCAACATTGTCATGTCTGTATCCAAC
CACGAAAAGGAAATGGCTGCCATGGCTAAGAGGTTGAACGCCAAAGAATTGCGTAAGGCACAGGCTGG
CCAGAGCGCTGAAATGAAACTCGCCAAAATTTCAATGGTAATTATTACCCAATTCATGCTCTCCTGGT
CTCCATACGCCATCATCGCTCTTCTTGCACAGTTTGGGCCAGCTGAATGGGTTACTCCTTATGCAGCT
GAATTGCCTGTCCTGTTTGCTAAAGCTTCAGCTATCCACAACCCAATTGTCTACTCTGTTTC
>mimus1
GACCTATGGCGGCATCCAAGAAAATGTCCCATAGAAGAGCTTTCCTCATGATTATCTTTGTGTGGATG
TGGTCCATTGTTTGGTCTGTCGGCCCCGTCTTCAACTGGGGAGCATACGTCCCTGAAGGTATTCTTAC
ATCTTGCTCTTTTGATTACCTGTCTACTGATTCTAGTACCAGATCTTTCATCTTGTGCATGTACTTCT
GTGGTTTCATGCTGCCCGTAGTTATCATCGCTTTCTGTTACTTCAACATTGTCATGTCTGTATCCAAC
CACGAAAAGGAAATGGCTGCCATGGCTAAGAGGTTGAACGCCAAAGAATTGCGTAAGGCACAGGCTGG
CCAGAGCGCTGAAATGAAACTCGCCAAAATTTCAATGGTAATTATTACCCAATTCATGCTCTCCTGGT
CTCCATACGCCATCATCGCTCTTCTTGCACAGTTTGGGCCAGCTGAATGGGTTACTCCTTATGCAGCT
GAATTGCCTGTCCTGTTTGCTAAAGCTTCAGCTATCCACAACCCAATTGTCTACTCTGTTTC

>mimus2
GACCTATGGCGGCATCCAAGAAAATGTCCCATAGAAGAGCTTTCCTCATGATTATCTTTGTGTGGATG
TGGTCCATTGTTTGGTCTGTCGGCCCCGTCTTCAACTGGGGAGCATACGTCCCTGAAGGTATTCTTAC
ATCTTGCTCTTTTGATTACCTGTCTACTGATTCTAGTACCAGATCTTTCATCTTGTGCATGTACTTCT
GTGGTTTCATGCTGCCCGTAGTTATCATCGCTTTCTGTTACTTCAACATTGTCATGTCTGTATCCAAC
CACGAAAAGGAAATGGCTGCCATGGCTAAGAGGTTGAACGCCAAAGAATTGCGTAAGGCACAGGCTGG
CCAGAGCGCTGAAATGAAACTCGCCAAAATTTCAATGGTAATTATTACCCAATTCATGCTCTCCTGGT
CTCCATACGCCATCATCGCTCTTCTTGCACAGTTTGGGCCAGCTGAATGGGTTACTCCTTATGCAGCT
GAATTGCCTGTCCTGTTTGCTAAAGCTTCAGCTATCCACAACCCAATTGTCTACTCTGTTTC
>oculifer
GACCTATGGCGGCATCCAAGAAAATGTCCCATAGAAGAGCTTTCCTCATGATTATCTTTGTGTGGATA
TGGTCCATTGTTTGGTCTGTCGGCCCCGTCTTCAACTGGGGAGCATACGTCCCTGAAGGTATTCTTAC
ATCTTGCTCTTTTGATTACCTGTCTACTGATCCTAGTACCAGATCTTTCATCTTGTGCATGTACTTCT
GTGGTTTCATGCTGCCCGTAGTTATCATCGCTTTCTGTTACTTCAACATTGTCATGTCTGTATCCAAC
CACGAAAAGGAAATGGCTGCCATGGCTAAGAGGTTGAACGCCAAAGAATTGCGTAAGGCACAGGCTGG
CCAGAGCGCTGAAATGAAACTCGCCAAAATTTCAATGGTAATTATTACCCAATTCATGCTCTCCTGGT
CTCCATACGCCATCATCGCTCTTCTTGCACAGTTTGGGCCAGCTGAATGGGTTACTCCTTATGCAGCT
GAATTGCCTGTCCTGTTTGCTAAAGCTTCAGCTATCCACAACCCAATTGTCTACTCTGTTTC
>ornatus
GACCTATGGCGGCATCCAAGAAAATGTCCCATAGAAGAGCTTTCCTCATGATTATCTTTGTGTGGATG
TGGTCCATTGTATGGGCTGTTGGCCCCGTTTTCAACTGGGGAGCCTACGTCCCTGAAGGTATCCTAAC
ATCTTGCTCTTTTGATTATCTGTCTACTGATTCTAGCACCAAATCTTTCATCTTGTGCATGTACTTCT
GCGGCTTCATGCTGCCCGTAGTTATCATCGCTTTCTGTTACTTCAACATTGTCATGTCTGTATCCAAC
CACGAAAAGGAAATGGCTGCCATGGCTAAGAGGTTGAACGCCAAAGAATTGCGTAAGGCACAGGCCGG
CCAGAGCGCTGAAATGAAACTTGCTAAAATTTCAATGGTAATTATTACCCAATTTATGCTTTCCTGGT
CTCCATACGCCATCATCGCTCTTCTTGCACAGTTTGGGCCAGCTGAATGGGTTACCCCATACGCAGCC
GAGTTGCCTGTCCTGTTTGCTAAAGGTTCAGCTATCCACAACCCAATTGTCTACTCTGTTTC
>vulgaris1
GACCTATGGCGGCATCCAAGAAAATGTCCCATAGAAGAGCTTTCCTCATGATTATCTTTGTGTGGATA
TGGTCCATTGTTTGGGCTGTCGGCCCCGTCTTCAACTGGGGAGCATACGTCCCTGAAGGTATTCTTAC
ATCTTGCTCTTTTGATTACCTGTCTACTGATTCTAATACCAGATCTTTTATCTTGTGCATGTACTTCA
TGGGTTTCATGCTGCCCGTAGTTATCATCGCTTTCTGTTACTTCAACATTGTCATGTCTGTATCCAAC
CACGAAAAGGAAATGGCTGCCATGGCTAAGAGGTTGAATGCCAAAGAATTGCGTAAGGCACAGGCTGG
CCAGAGCGCTGAAATGAAACTTGCCAAAATTTCAATGGTAATTATTACCCAATTCATGCTCTCCTGGT
CTCCATACGCCGTCATCGCTCTTCTTGCACAGTTTGGGCCAGCTGAATGGGTTACTCCTTATGCAGCT
GAATTGCCTGTCCTGTTTGCTAAAGCTTCAGCTATCCACAACCCAATTGTCTACTCTGTTTC
>vulgaris2
GACCTATGGCGGCATCCAAGAAAATGTCCCATAGAAGAGCTTTCCTCATGATTATCTTTGTGTGGATA
TGGTCCATTGTTTGGGCTGTCGGCCCCGTCTTCAACTGGGGAGCATACGTCCCTGAAGGTATTCTTAC
ATCTTGCTCTTTTGATTACCTGTCTACTGATTCTAATACCAGATCTTTTATCTTGTGCATGTACTTCA
TGGGTTTCATGCTGCCCGTAGTTATCATCGCTTTCTGTTACTTCAACATTGTCATGTCTGTATCCAAC
CACGAAAAGGAAATGGCTGCCATGGCTAAGAGGTTGAATGCCAAAGAATTGCGTAAGGCACAGGCTGG
CCAGAGCGCTGAAATGAAACTTGCCAAAATTTCAATGGTAATTATTACCCAATTCATGCTCTCCTGGT
CTCCATACGCCGTCATCGCTCTTCTTGCACAGTTTGGGCCAGCTGAATGGGTTACTCCTTATGCAGCT
GAATTGCCTGTCCTGTTTGCTAAAGCTTCAGCTATCCACAACCCAATTGTCTACTCTGTTTC
>vulgaris3
GACCTATGGCGGCATCCAAGAAAATGTCCCATAGAAGAGCTTTCCTCATGATTATCTTTGTGTGGATA
TGGTCCATTGTTTGGGCTGTCGGCCCCGTCTTCAACTGGGGAGCATACGTCCCTGAAGGTATTCTTAC
ATCTTGCTCTTTTGATTACCTGTCTACTGATTCTAATACCAGATCTTTTATCTTGTGCATGTACTTCA
TGGGTTTCATGCTGCCCGTAGTTATCATCGCTTTCTGTTACTTCAACATTGTCATGTCTGTATCCAAC
CACGAAAAGGAAATGGCTGCCATGGCTAAGAGGTTGAATGCCAAAGAATTGCGTAAGGCACAGGCTGG
CCAGAGCGCTGAAATGAAACTTGCCAAAATTTCAATGGTAATTATTACCCAATTCATGCTCTCCTGGT
CTCCATACGCCGTCATCGCTCTTCTTGCACAGTTTGGGCCAGCTGAATGGGTTACTCCTTATGCAGCT
GAATTGCCTGTCCTGTTTGCTAAAGCTTCAGCTATCCACAACCCAATTGTCTACTCTGTTTC

>vulgaris4
GACCTATGGCGGCATCCAAGAAAATGTCCCATAGAAGAGCTTTCCTCATGATTATCTTTGTGTGGATA
TGGTCCATTGTTTGGGCTGTCGGCCCCGTCTTCAACTGGGGAGCATACGTCCCTGAAGGTATTCTTAC
ATCTTGCTCTTTTGATTACCTGTCTACTGATTCTAATACCAGATCTTTTATCTTGTGCATGTACTTCA
TGGGTTTCATGCTGCCCGTAGTTATCATCGCTTTCTGTTACTTCAACATTGTCATGTCTGTATCCAAC
CACGAAAAGGAAATGGCTGCCATGGCTAAGAGGTTGAATGCCAAAGAATTGCGTAAGGCACAGGCTGG
CCAGAGCGCTGAAATGAAACTTGCCAAAATTTCAATGGTAATTATTACCCAATTCATGCTCTCCTGGT
CTCCATACGCCGTCATCGCTCTTCTTGCACAGTTTGGGCCAGCTGAATGGGTTACTCCTTATGCAGCT
GAATTGCCTGTCCTGTTTGCTAAAGCTTCAGCTATCCACAACCCAATTGTCTACTCTGTTTC
